# Reliability, Objectivity, Validity, and Reference Levels of the Austrian Pole Climbing Test (APCT)—A Novel Monitoring Tool for Assessing General Fitness in Children, Adolescents, and Young Adults

**DOI:** 10.3390/sports12090258

**Published:** 2024-09-18

**Authors:** Gerald Jarnig, Reinhold Kerbl, Mireille N. M. van Poppel

**Affiliations:** 1 Institute of Human Movement Science, Sport and Health, University of Graz, 8010 Graz, Austria; 2 Department of Pediatrics and Adolescent Medicine, LKH Hochsteiermark, 8700 Leoben, Austria

**Keywords:** sport motor test, strength endurance, reference value, fitness monitoring, climbing, talent scouting, children, adolescents, young adults

## Abstract

Climbing is an activity involving many major muscle groups and, therefore, it is suitable for assessing general physical fitness. The Austrian pole climbing test (APCT) was developed as a reliable and valid test for the assessment of general fitness levels in children, adolescents, and young adults. In this cross-sectional pilot study, 677 participants (aged 6 to 19 years) were assessed via the APCT. Subsequently, test quality criteria (reliability and objectivity) were assessed, and the test’s validity was evaluated through comparisons with other established fitness tests (hand grip strength, standing long jump, and pull- and push-ups). Additionally, age- and gender-specific reference values were generated. The reliability (ICC2.1 = 0.97, 95%CI 0.95 to 0.98) and objectivity (ICC2.1 = 0.99, 95%CI 0.99 to 0.99) of the APCT were found to be excellent. The APCT results correlated strongly with the hand strength per kilogram of body weight (right: r = 0.58; left: r = 0.53), number of pull-ups (with upper grip: r = 0.74; with lower grip: r = 0.69) and standing long jump (r = 0.61); a moderate correlation with the push-up test was observed (r = 0.44). The APCT is reliable, objective, and suitable for children, adolescents, and young adults with an affinity for sports. It offers a novel opportunity to assess fitness without time pressure, considering the anthropometric requirements.

## 1. Introduction

The World Health Organization (WHO) physical activity recommendations of one hour of moderate exercise per day are currently far from being achieved by many children and adolescents [1]; on the contrary, a worrying increase in sitting and screentime is observed in this age group [2,3,4,5]. The consequences of this trend are rising obesity rates [6] and associated health problems [7,8] that not only negatively affect individuals [9] but also have a significant negative socio-political and economic impact [10,11,12]. 

In contrast to the decline in general population movement [13], professional sport has developed into a billion-dollar business in recent years [14,15]—a business in which it is possible to rise from financial poverty to become a millionaire through physical activity and sufficient talent [16,17]. Talent scouting in particular is playing an increasingly important role in this billion-dollar business [18,19]. The search for exceptional sporting talent begins at an early stage in childhood by carrying out specific sports tests with the aim of discovering future sporting stars and providing them with optimum support in their development [18,19]. Depending on the sport, sport-specific tests are carried out in combination with an assessment of physical fitness [20,21].

Sports schools in Austria are environments where the WHO physical activity guidelines are practiced [22,23,24,25] and sports talents should be supported in the best possible way [26]. However, places at such schools are limited, which makes an athletic selection process necessary [27]; to accomplish this, a variety of sport-relevant parameters are assessed, and the best participants are offered a place at a sports school [27]. While there are standardized admission procedures for high schools in Austria (LEAA, Long-term Development Analysis of Athletes [28]—based on the Swiss “PISTE” system [29]), admission procedures for secondary schools differ depending on the location of the school; a frequently used test procedure is climbing a gymnastics pole, with the aim to climb up the pole as quickly as possible under time pressure (Table S1). This climbing under time pressure increases the risk of a grip error and the associated risk of falling and injury. Additionally, it must be noted that most of the tests use a published test manual with reference values, but do not take the children’s height or grip height into account [30,31].

Climbing is a sport that challenges people’s physical condition as a whole by using a large number of major muscle groups [32,33,34]. Based on high total body strength, climbing requires different physical conditions, such as high muscular endurance and a low body fat percentage [32]. This makes climbing an effective tool for assessing overall physical fitness quickly and easily [35,36].

The aim of this study was, therefore, to develop a valid, innovative novel pole climbing test for children, adolescents, and young adults, in which the anthropometric data of the participants are considered, and climbing speed is not relevant.

## 2. Materials and Methods

### 2.1. Study Design

A cross-sectional study was conducted at a school campus in Klagenfurt City, Austria, where three different types of school (primary school (average age 6 to 10 years), secondary school (average age 11 to 14 years), and high school (average age 15 to 19 years)) are situated in one building complex. The study was approved by the Research Ethics Committee of the University of Graz, Styria, Austria (GZ. 39/68/63 ex 2021/22).

All of the respective school directors agreed that their school would take part in the cross-sectional study. For the participation of individual pupils, the following inclusion criteria were defined: The children had to attend one of the three schools on campus and be able to complete all sport-specific tests without restrictions.

**Figure 1:**
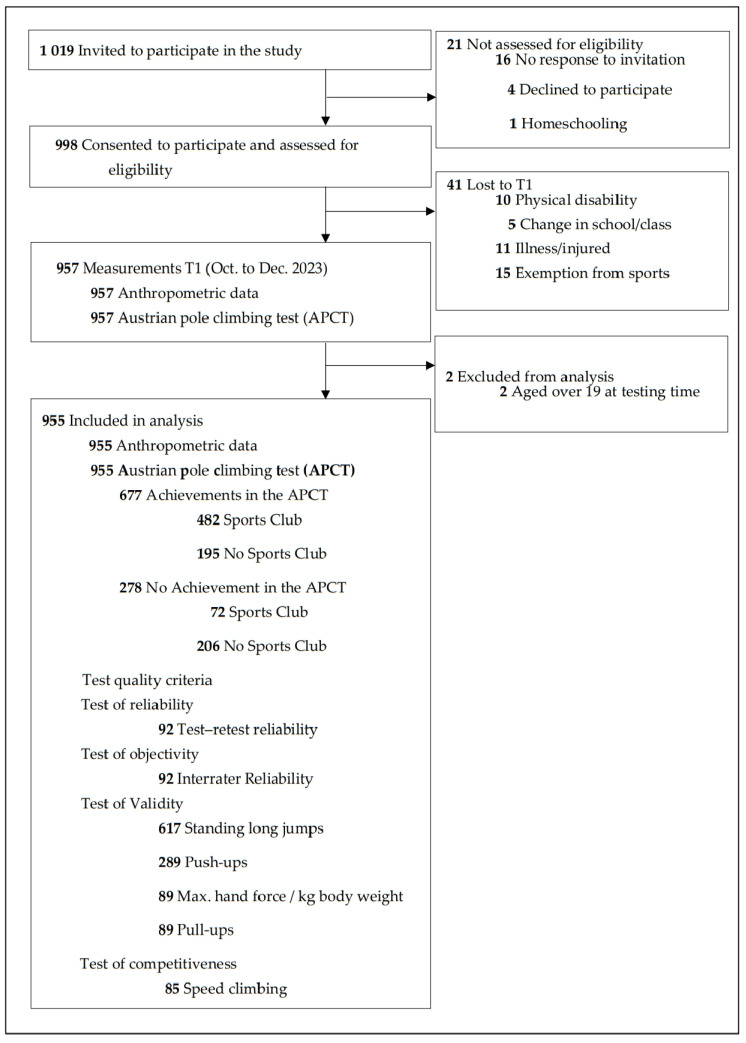
Flow diagram.

A total of 1019 children and adolescents were invited to take part in the study. The legal guardians of children aged 14 years and younger were informed in writing about the study content and asked for permission regarding their children’s participation. A total of 998 (97.9%) children and adolescents, and their respective legal guardians, consented to participate in the study and provided information regarding age, gender, and school type (Figure 1).

### 2.2. Procedure

The measurements of anthropometric data and sports motor tests were carried out by trained members of the research team and took place in the schools during sports classes over a two-week period. All tests were carried out with participants wearing standard sportswear but without shoes.

#### 2.2.1. Anthropometrics

Height (cm) was measured to the nearest 0.1 cm using an SECA 213 stadiometer, and weight (kg) was measured to the nearest 0.1 kg using a Bosch PPW4202/01 body scale. The body mass index (BMI) was calculated by dividing the body weight by the height in meters squared. Grip height was measured to the nearest centimeter using a measuring tape fixed on the wall. The participants stood on the ground and not wearing sports shoes, with one hand stretched vertically upwards; the maximum distance between the ground and the fingertips of the hand stretched upwards was measured.

#### 2.2.2. Austrian Pole Climbing Test (APCT)

**Figure 2:**
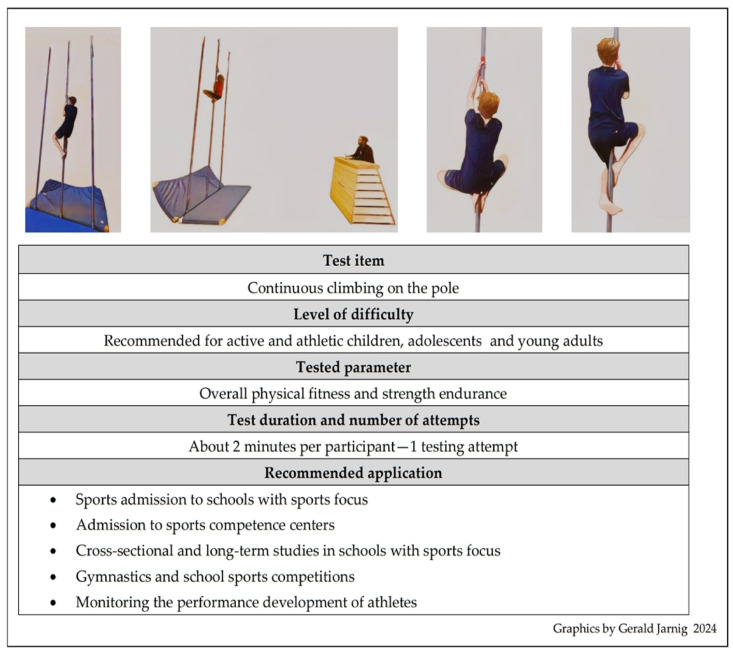
Key points of the Austrian pole climbing test.

The APCT is a fitness field test to assess general physical fitness and strength endurance in children, adolescents, and young adults (Figure 2).

Participants started from a fall protection mat and climbed continuously on a round, smooth climbing pole (steel bar ST35, ⌀ (42.5 ± 0.5) mm × (3.3 ± 0.1) mm) fixed at the top and bottom. Norm markings at heights of 2.0, 2.5, 3.0, 3.5, and 4.0 m from the floor were clearly visible on the climbing pole. The aim of the test is to climb over as many of these norm markings as possible within 2 min. The timespan of 2 min was chosen as it was described by participants in pilot tests as being perceived by them personally as reaching complete exhaustion. A norm marking was considered to have been climbed over as soon as the participant’s chin was above a norm marking. The participants could start a climbing attempt as often as they wanted within a 2 min period and take a break between climbing attempts according to their individual needs. There was no time limit for the last climbing attempt started within the 2 min time span; this last attempt could be carried out until physical exhaustion occurred. The highest norm markings climbed over in each individual climbing attempt were documented, and the overall climbing performance was calculated by summing the heights of each attempt, taking into account the grip height as well as the thickness of the fall protection mat.

Detailed information on the preparation, execution, documentation, calculation and evaluation of the APCT can be found in the Supplementary Materials (“Test Manual Austrian Pole Climbing Test (APCT)” (Additional Methods 2)).

#### 2.2.3. Strength Tests to Check Validity and Competitiveness

*Maximum hand force per kilogram body weight*

The participants’ maximum hand force in kilograms was measured using a hydraulic hand dynamometer (brand: Baseline; model number: 12-0241; product dimensions: 25.4 × 12.7 × 6.35 cm; 500 g). During the test, the participant sat with their shoulder abducted and neutrally rotated, their elbow bent at an angle of 90°, their forearm resting in a neutral position on a support (table), and the hand dynamometer held by hand; there was no contact between the support (table) and the hand dynamometer. To assess the maximum force, 3 maximum isometric contractions (each lasting 5 s) were performed with each hand (left and right), with a break of at least 1 min between each test. The maximum strength value of a hand was included in the overall assessment. The maximum hand force per kilogram of body weight was calculated by dividing the maximum hand force by the total body weight.

*Pull-ups (over- and underhand grip)*

Pull-ups were performed from a hanging position with both an overhand (main load on the triceps) and underhand grip (main load on the biceps) on a pull-up bar in the gym. The participants were asked to complete as many pull-ups as possible. A repetition was considered successfully completed if the chin of the test person moved above the pull-up bar and the body returned to the hanging position. The participants had one attempt, and the number of correctly completed pull-ups was included in the overall assessment.

*Push-ups*

The test was carried out according to the German motor test manual [37]. The participants lay on the floor in a prone position with their hands touching their glutes. To perform a push-up correctly, the hands were placed next to the shoulders, and the body was pushed upwards into a fully straight push-up position. In this position, one hand was released from the floor and had to touch the back of the other hand. The hand was then placed back on the floor and the body returned to the starting position in a controlled manner. The participants completed as many push-ups as possible within 40 s; the number of correctly completed push-ups was included in the analysis.

*Standing long jump*

The participants were positioned on a measuring mat for standing long jumps and jumped with both legs as far forward as possible from the starting line. The shortest distance between the starting line and the contact of the participant’s heels with the ground was measured on the mat to the nearest centimeter. Three evaluation attempts were performed, and the longest of the three jumps was included in the analysis.

*Speed Pole Climbing (SPC)*

The participants were asked to climb up the climbing pole as quickly as possible from an upright position. The time taken to reach the 4.0 m height norm mark with one shoulder or the highest norm mark reached was documented. A five-level categorization (1, very good, to 5, not enough) [30,31] was used based on existing reference values and included in the analysis.

### 2.3. Grouping, Standardization, and Classification

#### 2.3.1. Age Grouping

The participants were categorized into 7 age groups (≤7.9 years, 8.0 to 9.9 years, 10.0 to 11.9 years, 12.0 to 13.9 years, 14.0 to 15.9 years, 16.0 to 17.9 years, and 18.0 to 19.9 years), and gender-specific means and standard deviations were calculated for all the variables.

#### 2.3.2. Weight Classification

National reference values were used for the standardization of the BMI and the classification of weight [38]. More detailed information about the weight classification is given in the Supplementary Materials (Additional Methods 1).

#### 2.3.3. Development and Classification of Reference Values for the Austrian Pole Climbing Test (APCT)

Using the traditional z-score standardization mathematical formula [39], the climbing performance thresholds of −1.75, −1.25, −0.75, −0.25, +0.25, +0.75, +1.25, and +1.75 were calculated for the z-scores according to a nine-point rating scale (STA9) [40] in the different age groups and for both genders. Participants who had no achievement in the APCT (i.e., they were unable to climb the pole) were excluded from the calculation of the z-scores and the reference values.

$${APCT}_{STA9-CA}=\left({Z_{v}TV}_{STA9}\times {SD}_{age\;group\;\&\;sex}\right)-M_{age\;group\;\&\;sex}$$

Variables:

*APCT*_*STA*9*-CA*_ = Threshold values for performance in the Austrian pole climbing test according to a standardized nine-point rating scale.
*Z_v_TV_STA_*_9_ = Z-values of the traditional Z-standardization (threshold values: z = −1.75, z = −1.25, z = −0.75, z = −0.25, z = +0.25, z = +0.75, z = +1.25 and z = +1.75) according to a nine-point rating scale.
*SD_age group & sex_* = age- and gender-specific standard deviations.
*M_age group & sex_* = age- and gender-specific mean values.

A nine-point classification was developed using the calculated threshold values in the different age groups and for both genders. The worst sporting performance was classified with 1 point and the best performance was classified with 9 points (Table S2) [41].

### 2.4. Test Criteria

#### 2.4.1. Main Test Criteria: Reliability, Objectivity, and Validity

To assess the test–retest reliability of the APCT, the test participants were tested twice by the same test administrator (intrarater reliability), with an interval of between one and two weeks between the test days.

Objectivity was assessed using interrater reliability, whereby each participant’s performance at the APCT was observed, documented, and evaluated by two test administrators simultaneously and independently of each other.

In addition to climbing technique, the key performance factors in pole climbing are strong hands, arms, legs, and trunk, so the best possible total body strength and strength endurance are the key to achieving top results [42]. For this reason, different strength tests (a test of the maximum force of the hands using a dynamometer [43], a standing long jump (considered an indicator of total body strength) [44], and strength endurance tests (pull-up tests, upper and lower grip; push-up test)) were selected, and the data of the APCT performances and those of the strength tests were correlated in order to assess validity.

#### 2.4.2. Secondary Quality Test Criteria

In order to assess the competitiveness of the APCT, the results of an established pole climbing field test (SPC) [30,31] and the APCT were categorized and compared with each other.

#### 2.4.3. Selection of Participants for Review of Test Quality Criteria and Competitiveness

The reliability, objectivity, competitiveness, and aspects of validity (maximum hand force and pull-ups) were tested in children in the secondary school sports branch for whom it was organizationally possible to repeat these tests within two weeks or for whom two test administrators could carry out the tests simultaneously.

### 2.5. Statistical Analysis

Continuous variables are reported as the mean (M) and standard deviation (SD), and categorical variables are reported as absolute numbers (n) and percentages (%). The APCT and SPC data were visually checked for normal distributions.

To identify differences between groups, unpaired *t*-tests and chi-square tests were performed; in the absence of homogeneity of variance, the Welch test was performed; a Mann–Whitney U test was used to assess the differences in weight categories, and the Games–Howell post hoc test was used to assess the development of APCT performance between the gender-specific age categories.

For both test–retest and interrater reliability, a two-sided mixed intraclass correlation coefficient (ICC) was calculated based on individual measures and absolute agreement for the APCT raw scores [45]. To determine the reliability and objectivity, the 95% CIs of the ICCs were interpreted as follows: <0.5 = poor; 0.5 to 0.75 = moderate; 0.75 to 0.90 = good; >0.90 = excellent reliability or objectivity.

To assess validity and competitiveness, the Pearson correlation coefficient (r) was employed to calculate the correlation between the APCT performance and the results of the strength tests or SPC. The strength of correlations was classified according to Cohen [46], whereby a weak correlation was classified as r ≥ 0.1, a moderate correlation as r ≥ 0.3, and a strong correlation as r ≥ 0.5.

No imputation of data was performed. All the statistical analyses were performed in SPSS 29.0 (IBM SPSS Statistics 29, IBM, New York, NY, USA) with a significance level of *p* < 0.05.

## 3. Results

Between September and December 2023, a total of 957 children and adolescents took part in the anthropometric and sports motor measurements (due to a lack of resources, detailed results of the anthropometrics and sports assessments are reported in the Supplementary Materials). Two participants, who were older than 19 years at the time of the measurements, were excluded from the analysis (Figure 1).

Of the remaining 955 participants, 278 (29.1%) showed no achievement in the APCT. These participants were significantly more often in the higher weight categories (overweight, obese, and extremely obese) compared to participants with achievements in the APCT (Figure 1, Tables S3 and S4). A significant (*p* < 0.001) trend was observed wherein more boys showed achievements in the APCT than girls. This trend was highly significant (*p* < 0.001) for the age groups from 14 to 19 years. Additionally, significant differences were found in the age groups from 10 to 13 years. No significant differences were observed between girls and boys in the youngest age groups (school entry to 9 years) (Figure S1, Table S5).

The APCT raw data showed a continuous increase in mean performance for boys up to the age category of 16 to 17 years old, after which the mean performance decreased slightly (*p* > 0.99). In girls, the mean APCT performance in the different age categories increased up to the age category of 12 to 13 years old, after which a continuous albeit non-significant decrease in performance was observed (Figure S2, Tables S6–S8).

The gender- and age-specific reference values calculated using the threshold values of a standardized nine-point rating scale are presented in Table 1 (see also Table S9).

The assessment of reliability and objectivity showed a high degree of confidence. The reliability of all the participants was excellent (ICC = 0.97 (95% CI, 0.95–0.98)); this was observed in boys (ICC = 0.97 (95% CI, 0.95–0.98)) as well as girls (ICC = 0.96 (95% CI, 0.95–0.98)). Objectivity was excellent for all participants (ICC = 0.99 (95% CI, 0.99–0.99)) as well as in both genders (Table 2, Tables S10 and S11).

The testing of validity showed strong correlations between APCT achievements and maximum hand force per kilogram of body weight (right hand: r = 0.58; left hand: r = 0.53), pull-up variations (PU-OG = 0.74; PU-UG = 0.69), and the standing long jump (SLJ = 0.61). A moderate correlation was found between achievements in APCT and push-ups (r = 0.44). Comparable results were observed in boys and girls (Table 3, Tables S12 and S13).

Finally, a check of the competitiveness with a frequently used SPC test shows a strong negative correlation (−0.59) of the raw data between APCT and SPC across all participants; the same trend is observed for boys and girls (Table S14). The real climbing height completed was 2.01 m on average per climbing attempt (range: 0.75 m (1.68 m to 2.43 m)) (Table S6), and a visual check between the classification of both pole climbing field tests (APCT vs. SPC) for normal distribution shows a more satisfactory result without a pronounced ceiling effect in the APCT (Figure S3).

## 4. Discussion

A high percentage of participants (29.1%) were not able to perform the APCT, which indicates that the APCT is not suitable for use in the general population of children and adolescents. This is hardly surprising, especially considering the fact that a general decline in physical activity among children and young people is being observed worldwide [13,47,48]. Pole climbing, like climbing in general, requires a very specific movement that is both physically and mentally challenging [32]. For children who do not participate in regular sports in their leisure time, this type of athletic performance can, therefore, often present an unmanageable challenge. However, it might be a useful test in the selection of sporting talent. This is in line with the current use of other pole climbing tests, which are primarily used for selection in the field of sport (Table S1). The observed reliability and objectivity of the APCT indicate a high level of confidence in the measurement method, thus confirming that this new field test can also be used in long-term studies with the aim of assessing the general fitness of children, adolescents, and young adults. The high correlations between APCT performance and the data for the maximum hand strength per kilogram of body weight, pull-up tests (upper and lower grip), and standing long jump (considered as an index for the general assessment of muscular fitness in children [44]) results suggest a high validity in assessing the whole body muscular fitness level of the participants. This finding is further supported by the data from the push-up test (moderate correlation).

In another pole climbing test, the climber needs to climb up the pole as fast as possible [31]. The grip height is not given any consideration in this test; other test manuals used (Table S1) are described only in the gray literature, and offer no reference values, without a verification of reliability, objectivity, or validity.

The novel APCT offers the possibility of assessing pole climbing by taking anthropometrics into account. We can see from the data of the SPC that the real climbing height per climbing attempt varied between 1.68 m and 2.43 m, which means that the participant with the lowest grip height has to climb 44.6% further in order to be able to complete the full height of the pole, an aspect that makes the results of a test assessment not taking the grip height into account questionable. In addition, SPC involves climbing under enormous time pressure, which can lead to an increased risk of injury and falls, factors which may be arguments for using the APCT in the future when assessing climbing performance on the gymnastics pole.

The novel APCT offers further benefits. In the course of a school year, young athletes contact those responsible at sports schools for varying reasons (change in family circumstances, relocation, change in club, etc.) and ask for admission to the ongoing sports activities. In addition to a sport-specific commitment from those responsible for their sports clubs, the athlete’s physical aptitude must also be checked. In this context, the APCT offers a practicable general alternative to a variety of fitness tests. The comparison with strength and strength endurance tests showed strong correlations between the test results of these parameters and the results of the APPCT, making the APCT suitable for providing a quick assessment of the test participant’s general physical fitness.

One of the advantages of the APCT is that it is easy to perform, which means less complexity for the test participants. In addition, the test’s quick execution saves important time, which reduces testing costs and can lead to an increase in motivation among test participants. As participants only have to mentally prepare for one test, this can reduce their stress and allow them to focus fully on performing this test. In addition, the likelihood of errors is reduced as there are fewer opportunities for measurement errors or misunderstandings.

The results of the test quality criteria show very strong reliability values, which can be seen as a positive aspect of practicability. The simple and rapid test implementation enables and simplifies its use in testing large groups of athletes.

Of course, there are alternative ways of assessing the general physical performance of participants. However, these requires a complex structure, as is the case in various standardized fitness courses, such as the frequently used obstacle run course [49]. Another way of assessing whole-body fitness consists in using existing test batteries consisting of a number of different individual tests, as for example EUROFIT [50], the German Motor Test [37], or the FitnessGram [51].

The APCT, therefore, offers numerous advantages, including time and cost savings, simpler implementation, faster results, less participant tiredness and stress, and increased motivation and willingness to participate in the test. It reduces complexity, susceptibility to errors, and psychological stress, while at the same time scoring positively with high comparability, availability, and flexibility, and is particularly suitable for large groups and spontaneous testing.

### Strengths and Limitations

One of the strengths of this study is that the assessment of the test quality criteria was carried out with a large number of participants. Another strength is that for the first time in a pole climbing test manual, the grip height of the test subject was included in the assessment. In addition, the age- and gender-specific reference values presented enable the use and assessment of APCT performance in future studies. Finally, the detailed test manual in the Supplementary Materials (Additional Methods 2) provides an easy-to-use tool for the selection of pupils in sports schools.

This study has some limitations, such as the high percentage of participants not able to perform the test in a general school, the low number of participants in each age group, the lack of annual age groups in the results, and the fact that all data were collected at one location in Austria. Reference values must, therefore, be used with these details in mind.

## 5. Conclusions

The novel APCT proposed in this study includes grip height when testing pole climbing, making the results valid and reliable. Furthermore, not using climbing speed as a criterion reduces the risk of injuries. Although climbing poles are part of the basic equipment in many gyms and sports halls, and strong correlations with strength and strength endurance tests have been demonstrated, tests based on pole climbing are rarely reported in publications. The novel APCT could give way to more and better evaluation of climbing skills in sports schools. Further widespread studies should be carried out to generate representative reference values for different age groups (at one-year intervals).

## Figures and Tables

**Table 1:** Age- and gender-specific reference values for the Austrian pole climbing test.

Variable	Gender	Age Group	Categories of Performance According to the Austrian Pole Climbing Test								
			1	2	3	4	5	6	7	8	9
Reference values [m]	Male	≤7	≤0.00	0.01–0.40	0.41–1.37	1.38–2.35	2.36–3.32	3.33–4.29	4.30–5.26	5.27–6.23	>6.23
		8 to 9	≤0.00	0.01–0.43	0.44–2.02	2.03–3.60	3.61–5.19	5.20–6.78	6.79–8.37	8.38–9.95	>9.95
		10 to 11	≤0.00	0.01–0.68	0.69–2.59	2.60–4.51	4.52–6.42	6.43–8.34	8.35–10.26	10.27–12.17	>12.17
		12 to 13	≤0.00	0.01–1.95	1.96–4.15	4.16–6.36	6.37–8.56	8.57–10.77	10.78–12.97	12.98–15.18	>15.18
		14 to 15	≤0.00	0.01–2.96	2.97–5.32	5.33–7.68	7.69–10.03	10.04–12.39	12.40–14.75	14.76–17.11	>17.11
		16 to 17	≤0.00	0.01–2.77	2.78–5.58	5.59–8.39	8.40–11.20	11.21–14.00	14.01–16.81	16.82–19.62	>19.62
		18 to 19	≤0.00	0.01–5.33	5.34–7.02	7.03–8.71	8.72–10.40	10.41–12.09	12.10–13.77	13.78–15.46	>15.46
	Female	≤7	≤0.00	0.01–0.21	0.22–1.00	1.01–1.78	1.79–2.57	2.58–3.35	3.36–4.14	4.15–4.93	>4.93
		8 to 9	≤0.00		0.01–1.53	1.54–4.00	4.01–6.48	6.49–8.95	8.96–11.42	11.43–13.90	>13.90
		10 to 11	≤0.00		0.01–2.00	2.01–4.21	4.22–6.42	6.43–8.63	8.64–10.83	10.84–13.04	>13.04
		12 to 13	≤0.00	0.01–0.17	0.18–2.44	2.45–4.72	4.73–6.99	7.00–9.27	9.28–11.54	11.55–13.82	>13.82
		14 to 15	≤0.00	0.01–0.16	0.17–2.07	2.08–3.98	3.99–5.89	5.90–7.80	7.81–9.71	9.72–11.62	>11.62
		16 to 17	≤0.00	0.01–0.16	0.17–1.86	1.87–3.56	3.57–5.26	5.27–6.97	6.98–8.67	8.68–10.37	>10.37
		18 to 19	≤0.00	0.01–0.49	0.50–1.64	1.65–2.78	2.79–3.93	3.94–5.08	5.09–6.22	6.23–7.37	>7.37
Categories of classification											
Low c.p.				Average c.p.				High c.p.			
1	No c.p.			4	Below-average c.p.			7	Good c.p.		
2	Poor c.p.			5	Average c.p.			8	Very good c.p.		
3	Weak c.p.			6	Above-average c.p.			9	Excellent c.p.		

c.p. = climbing performance.

**Table 2:** Reliability and objectivity of APCT performance for all and separately for boys and girls.

Test Quality Criteria	Age Group	Group	N	ICC (2.1)	95% CI		Agreement
					Lower	Upper	
Test of Reliability	All	All	92	0.968	0.952	0.979	Excellent
		Boys	68	0.972	0.954	0.982	Excellent
		Girls	24	0.959	0.909	0.982	Excellent
Test of objectivity	All	All	92	0.999	0.999	0.999	Excellent
		Boys	68	0.999	0.999	0.999	Excellent
		Girls	24	0.999	0.998	>0.999	Excellent

Explanation: To determine reliability (test [APCT R1 T1] and retest [APCT R1 T2]) and objectivity (test rater 1 [APCT R1 T1] and test rater 2 [APCT R2 T1]), the 95% CIs of the ICCs were interpreted as follows: 95% CI values below 0.5 were considered to indicate poor reliability, values between 0.5 and 0.75 were considered to indicate moderate reliability, values between 0.75 and 0.9 were considered to indicate good reliability, and values above 0.90 were considered to indicate excellent reliability. ICC = intraclass correlation; CI = confidence interval; APCT = Austrian pole climbing test; T1 = baseline measurements made in autumn 2023; T2 = measurement taken within 2 weeks of the baseline measurement; R1 = rater 1; R2 = rater 2.

**Table 3:** Checking for validity using Pearson’s correlation (r) coefficient.

Age Group	Upper Body Strength						Lower Body Strength		
	Maximum Hand Force, Pulling Force Endurance of the Arms			Pushing Force Endurance of the Arms and Core Stability			Muscle Strength of the Legs		
	Group	Variable	APCT R1 T1, rs	Group	Variable	APCT R1 T1, rs	Group	Variable	APCT R1 T1, rs
All	All (N = 89)	MHF—R, kg/kg-bw	0.579 **	All (N = 289)	Push-ups, No.	0.443 **	All (N = 617)	SLJ, cm	0.611 **
		MHF—L, kg/kg-bw	0.530 **						
		PU—OG, No.	0.743 **						
		PU—UG, No.	0.691 **						
	♂ (N = 66)	MHF—R, kg/kg-bw	0.623 **	♂ (N = 180)	Push-ups, No.	0.410 **	♂ (N = 412)	SLJ, cm	0.616 **
		MHF—L, kg/kg-bw	0.596 **						
		PU—OG, No.	0.752 **						
		PU—UG, No.	0.692 **						
	♀ (N = 23)	MHF—R, kg/kg-bw	0.548 **	♀ (N = 109)	Push-ups, No.	0.495 **	♀ (N = 205)	SLJ, cm	0.452 **
		MHF—L, kg/kg-bw	0.444 *						
		PU—OG, No.	0.822 **						
		PU—UG, No.	0.818 **						

Data are Pearson’s correlation coefficient (r): The strength of the correlations was classified according to Cohen, whereby a weak correlation was classified as ≥0.1, a moderate correlation as ≥0.3, and a strong correlation as ≥0.5; * = correlation is significant at the 0.05 level (2-tailed); ** = correlation is significant at the 0.01 level (2-tailed); kg = kilogram; cm = centimeter; kg/kg-bw = kilogram per kilogram of body weight; m = meter; T = measurement time point; MHF—R = maximum hand force, right hand; MHF—L = maximum hand force, left hand; PU—OG = pull-ups, over-grip; PU—UG = pull-ups, under-grip; APCT = Austrian pole climbing test; ♂ = boys; ♀ = girls.

## Data Availability

The data presented in this study are available on request from the corresponding author. The data are not publicly available due to privacy/ethical restrictions.

## Supplementary Materials

The following are available online at https://www.mdpi.com/article/10.3390/sports12090258/s1, Additional Methods 1: Weight classification; Additional Methods 2: Test manual—Austrian pole climbing test; Table S1. Application in Austria of pole climbing in selection procedures; Table S2. Classification of the Austrian pole climbing test; Table S3. Differences between participants with and without achievements in the Austrian pole climbing test; Table S4. Mann–Whitney U test used to assess differences in classification groups between participants with and without achievements in the Austrian pole climbing test; Table S5. Differences between participants with and without APCT achievements in age- and gender-specific subgroups; Table S6. Overall sample characteristics; Table S7. Gender- and age-specific mean values of participants with achievements in the Austrian pole climbing test; Table S8. Multiple comparisons between the gender-specific age categories for differences in achievement in the APCT using the Games–Howell post hoc test; Table S9. Performance thresholds for APCT defined by thresholds of traditional z-score standardization (−1.75, −1.25, −0.75, −0.25, 0.25, 0.75, 1.25, and 1.75) for the classification of a nine-point assessment (according to data from our own study population); Table S10. Descriptive statistics of the results for checking reliability and objectivity; Table S11. Reliability and objectivity of APCT performance in different age groups and as well separately for boys and girls; Table S12. Descriptive statistics of the results for checking validity; Table S13. Age-specific data from the validity check using Pearson’s correlation coefficient; Table S14. Checking competitiveness using Pearson’s correlation coefficient; Figure S1. Achievement versus non-achievement in the Austrian pole climbing test according to the total study population and for the subgroups of boys (♂) and girls (♀); Figure S2. Gender- and age-specific mean Austrian pole climbing test raw values; Figure S3. Graphical checking of normal distribution using histograms for the classification of the Austrian pole climbing test and an established speed pole climbing test (further reference in supplements [52]).
