# Supplementary material for: Reliability, Objectivity, Validity, and Reference Levels of the Austrian Pole Climbing Test (APCT)—A Novel Monitoring Tool for Assessing General Fitness in Children, Adolescents, and Young Adults
Source: Sports (Basel). 2024 Sep 18;12(9):258. doi: 10.3390/sports12090258 (PMC11435566; doi:10.3390/sports12090258)
Supplement: Supplementary file 1 [file sports-12-00258-s001.zip › sports-3186540-supplementary.pdf]

Reliability, Objectivity, Validity, and Reference Levels of the Austrian Pole Climbing Test (APCT)—A Novel Monitoring Tool for Assessing General Fitness in Children, Adolescents, and Young Adults

Supplementary material

This supplementary material has been provided by the authors to give readers additional information about their work.

Content:

Additional Methods 1: Weight classification

Additional Methods 2: Test – Manual – Austrian Pole Climbing Test

Table S1. Application in Austria of pole climbing in selection procedures

Table S2. Classification of the Austrian Pole Climbing Test

Table S3. Differences between participants with and without achievements at the Austrian Pole Climbing Test

Table S4. Mann-Whitney U-test used to assess differences in classification groups between participants with and without achievements at the Austrian Pole Climbing Test

Table S5. Differences between participants with and without APCT achievements in age and gender-specific subgroups

Table S6. Overall sample characteristics

Table S7. Gender and age-specific mean values of participants with achievements at the Austrian Pole Climbing Tests

Table S8. Multiple comparisons between the gender-specific age categories for differences in achievement at APCT using the Games-Howell post-hoc test.

Table S9. Performance thresholds for APCT defined by thresholds of traditional z-score standardization (-1.75, -1.25, -0.75, -0.25, 0.25, 0.75, 1.25 and 1.75) for the classification of a nine-point assessment (according to data from the own study population)

Table S10. Descriptive statistics of the results for checking reliability and objectivity

Table S11. Reliability and objectivity of APCT performance in different age groups and as well separately for boys and girls

Table S12. Descriptive statistics of the results for checking validity

Table S13. Age-specific data from the validity check using Pearson's 's correlation coefficient

Table S14. Checking competitiveness using Pearson's 's correlation coefficient

Figure S1. Achievements versus No- Achievements at the Austrian Pole Climbing Test by the total study population and for subgroups boys (♂) and girls (♀)

Figure S2. Gender and age-specific mean values of raw values of the Austrian Pole Climbing Test

Figure S3. Graphical checking of normal distribution using histograms for classification of Austrian Pole Climbing Test and an established Speed Pole Climbing Test.

Additional Methods 1: Weight classification

National reference values were used for the standardization of the BMI and the classification of weight. [1] The national reference data were expressed in BMI centile curves (i.e. equicurves, called EQUI BMI in this report). [1] The absolute BMI values were converted to EQUI BMI values according to the procedure described in Mayer et al. [1] (based on Cole et al. [2]). The EQUI-BMI curves were used to project the actual BMI to cut-off values at 18 years of age to categorize the children's weight into five categories (underweight < 18.5 kg/m2, normal weight = 18.5 to 25.0 kg/m2, overweight ≥ 25.0 kg/m2, obese ≥ 30.0 kg/m2, morbid obesity ≥ 35.0 kg/m2). For participants aged > 18 years, the crude BMI was used to assign a weight classification.

TEST MANUAL

Test organization

Assessing the general fitness and strength endurance of participants in the gym and sports hall by climbing-on-climbing poles

Test item

The test item is designed to test and assess general fitness and strength endurance. The test person completes a series of climbing attempts on the climbing pole, depending on their individual ability. The aim is to reach the highest possible total climbing height in 2 minutes.

Test item:	Climbing on the climbing pole
Test level:	Recommended for children, adolescents and young adults with an affinity for sport
Test parameter:	General fitness and strength endurance
Test duration:	2 minutes
Test intensity:	High to maximum physical activity
Test assessment:	Total climbing height by taking into account the grip height of participants

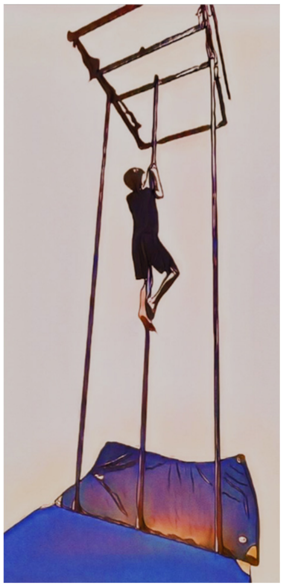

Austrian Pole Climbing Test for assessing general fitness and strength endurance in children, adolescents and young adults - Graphics by Gerald Jarnig 2024

Test material

Two gym mats, climbing poles with norm marks and a stopwatch (cell phone or handheld stopwatch) are required. If the climbing poles do not have norm marks, a marking tape is also required.

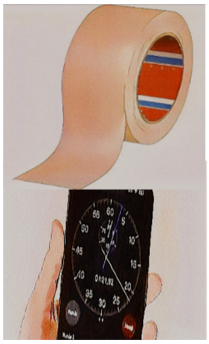

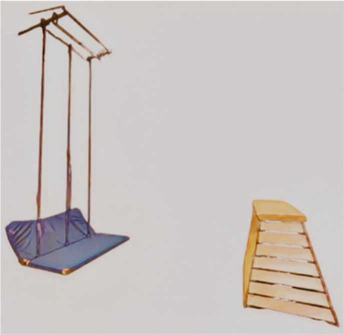

Requirements for the Austrian Pole Climbing Test Graphics by Gerald Jarnig 2024

Test staff
A test administrator can assess a maximum of 2 participants simultaneously.

Test construction
Setup of a test station: Climbing poles are fixed at the top and bottom in accordance with regulations and the surrounding area is protected with gym mats. If there are no norm marks on the climbing poles, these are marked at heights of 2.0 meters, 2.5 meters, 3.0 meters, 3.5 meters and 4.0 meters using a marking tape.

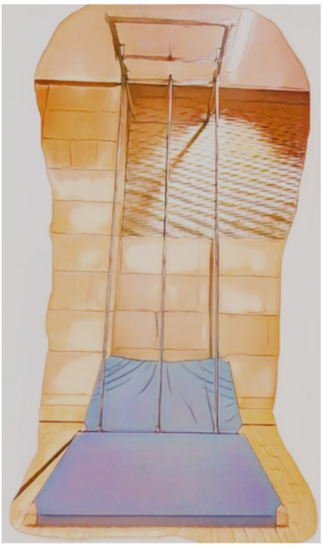

Setup of the Austrian Pole Climbing Test Station - Graphics by Gerald Jarnig 2024

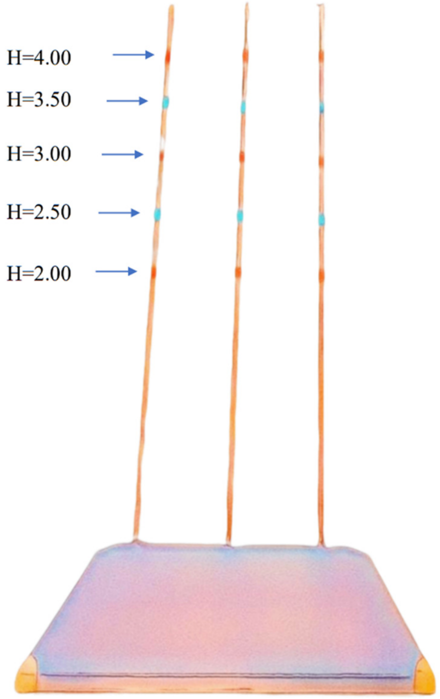

Norm marks
Austrian
Pole Climbing Test
(dimensions in m)

Graphics by Gerald Jarnig 2024

<u>**Note:**</u> According to Ö-Norm S 4622 (Edition:2021-10-15) "Gymnastics equipment - Climbing poles", there are climbing poles with norm marks up to a height of 4.50 meters.

Safety gym mats
Depending on local conditions, the ground around the climbing station must be made safe with gym mats to prevent falls and uncontrolled descent. The strength/thickness of the safety mat from which the test participants start the climbing attempt must be documented on the test assessment sheet and is taken into account when calculating the total climbing height.
Note: Depending on the weight of the test participants, the safety mat is compressed to varying degrees when the participants step on it. For mats with a thickness of over 20 cm (uncompressed state), this is taken into account with a mat reduction thickness of 5 cm in the final calculation of the total climbing height; for mats with a thickness of up to 20 cm (uncompressed state), this is not taken into account.
Different types of protection:

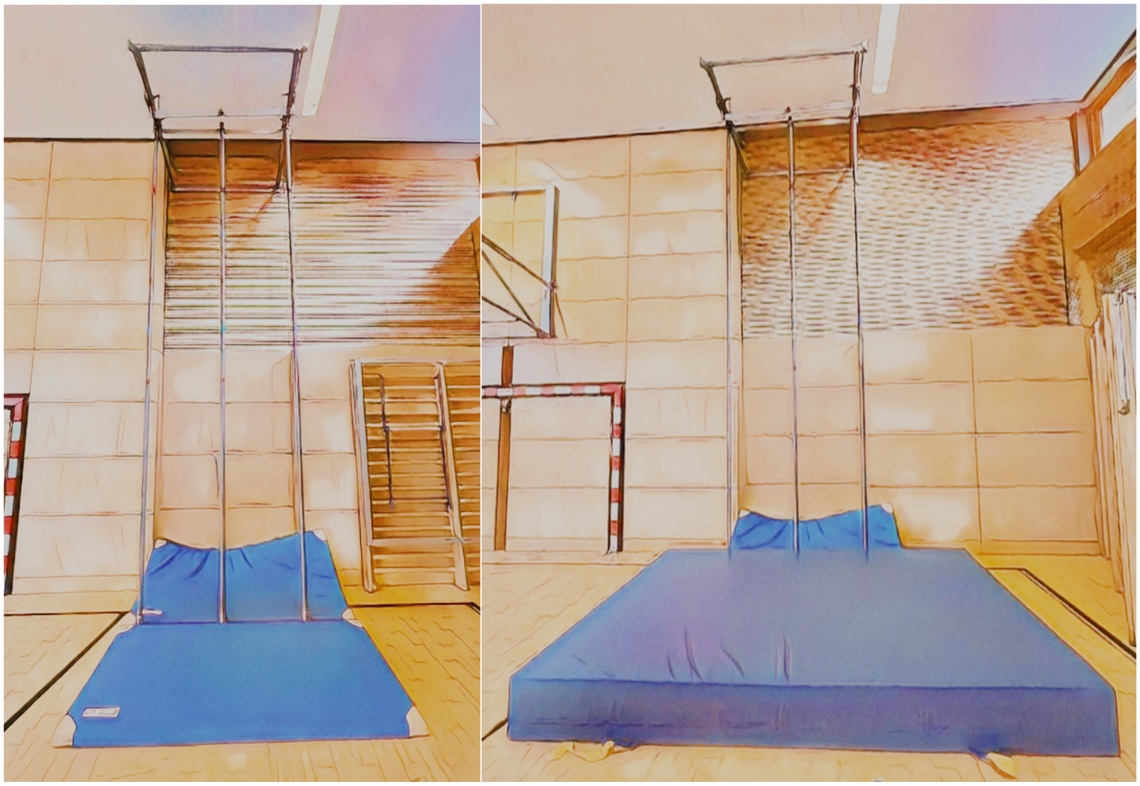

For mat thicknesses up to 20 cm, the compression of the mat by the participant is not taken into account in the calculation.

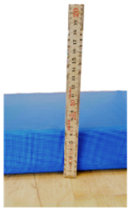

For mat thicknesses over 20 cm, the compression of t[illegible] mat by the participant is tak[illegible] into account in the calculati[illegible]

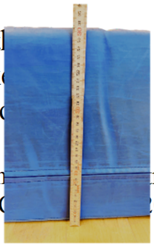

Variations of the safety gymnastic m[illegible]rian Pole Climbing Test Graphics by [illegible]024

Test administrator station

The test administrator is positioned at a distance of at least 4.00 m, centrally opposite the climbing station (a gymnastics box can be used as a writing surface).

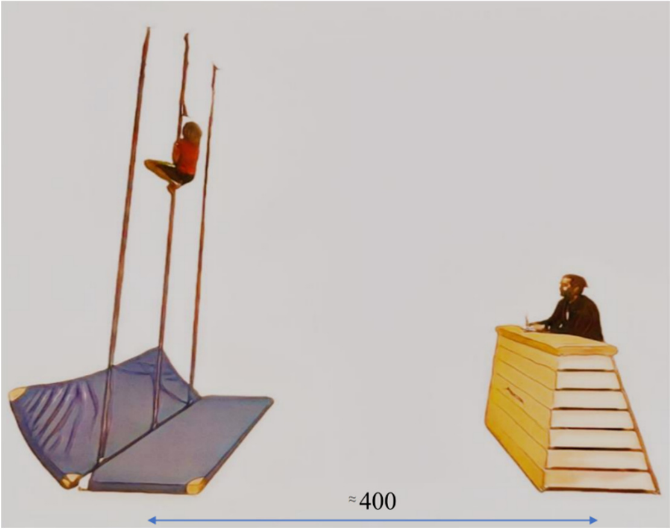

Test administrator station of the Austrian Pole Climbing Test (dimensions in cm) - Graphics by Gerald Jarnig 2024

Test procedure

The test is based on a series of climbing attempts on the climbing pole; as many climbing attempts as possible should be started within a period of 2 minutes and the highest possible total climbing height should be achieved.

Test preparation

The test administrator demonstrates the climbing technique to the participants and shows execution errors that increase the risk of injury and are therefore forbidden and lead to an early termination of a test attempt.
The participants should try out the climbing technique (max. 5 to 10 seconds) and the test administrator corrects any errors of execution. In addition to the anthropometric measurements of height and weight, the maximum grip height (in cm) in an upright position is measured for each participant.

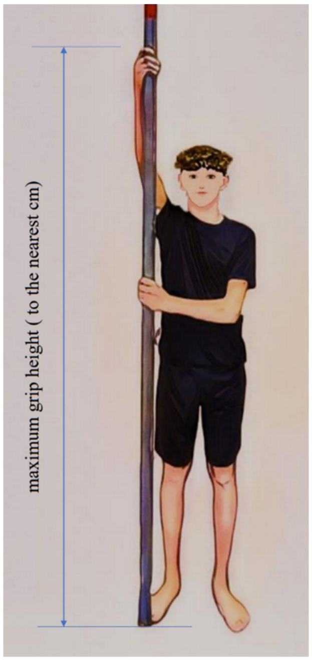

Measuring the grip height (dimensions in cm) - Graphics by Gerald Jarnig 2024

Climbing technique

Climbing technique in general:	By gripping the arms upwards (pulling) and then pushing the legs forward, the holding function when climbing on the pole is continuously taken over alternately by the arms and legs. Both legs form a climbing lock, whereby two permissible variants can be used. The holding function results in alternating pushing (with the legs) and pulling (with the arms) movements. The upper body and torso are as close as possible to the pole.
Legs holding technique Variant 1:	① The left and right instep side and parts of the soles of the feet are pressed against the pole.
Legs holding technique Variant 2:	② The leg in front of the pole is pressed against it with the sole or heel and the inside of the calf, while the outside of the foot, the inside of the shin and the inside of the knee press the leg behind the pole against it.
Arm holding technique:	③ The arms are held on the pole by means of a double grip.

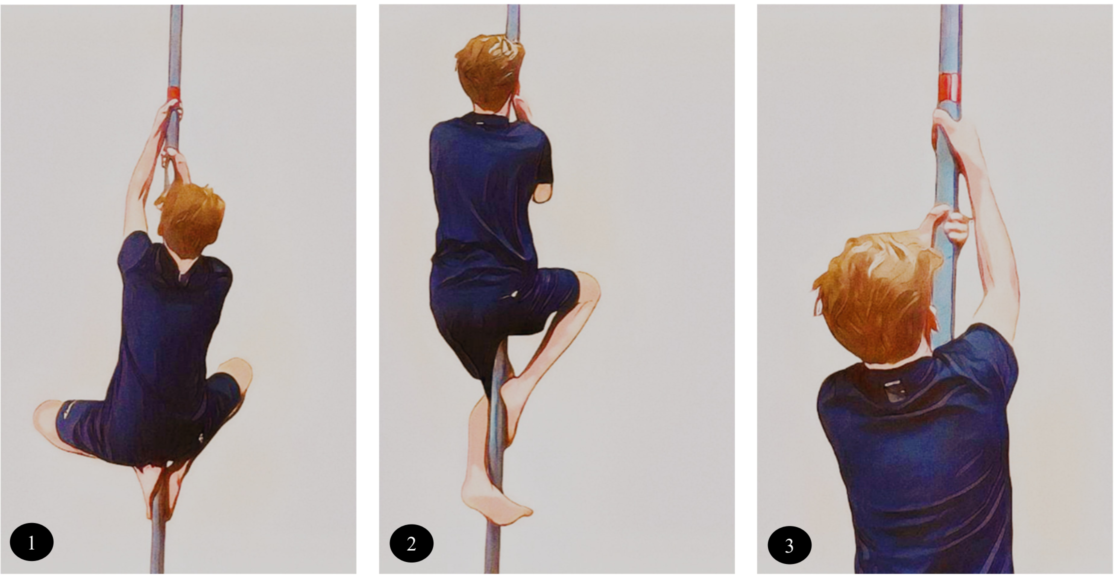

① Leg hold technique variant 1
② Leg hold technique variant 2
③ Arm holding technique

Graphics by Gerald Jarnig 2024

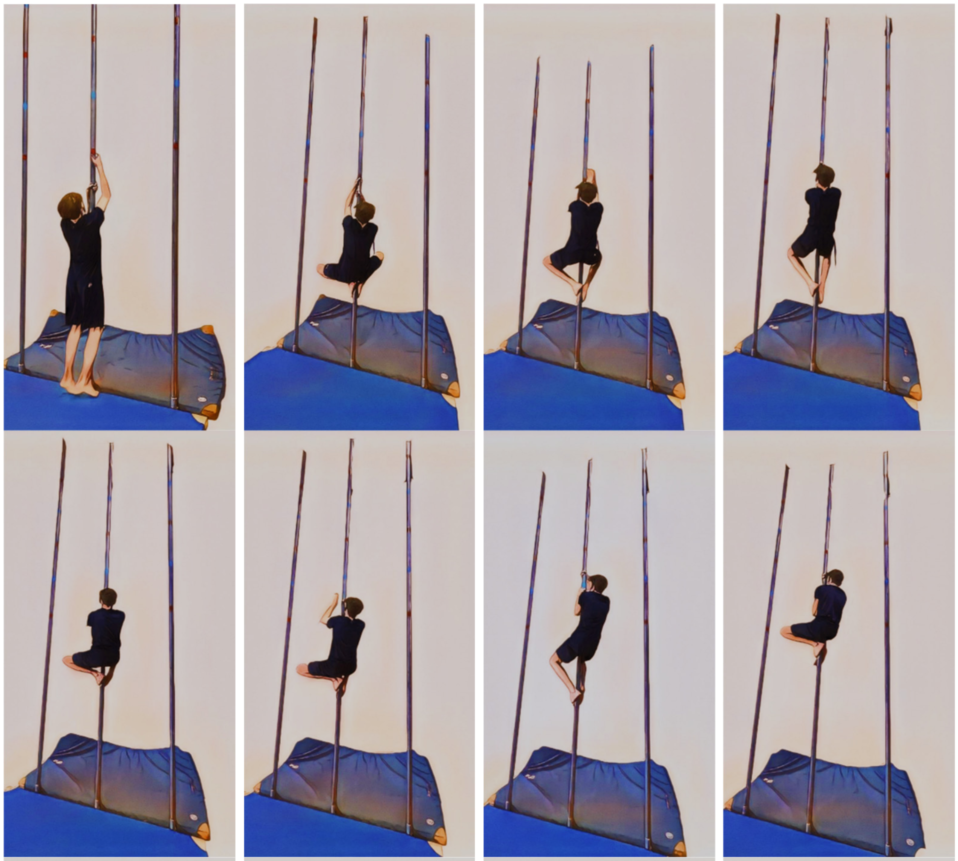

Climbing technique Austrian Pole Climbing Test - Leg holding technique variant 1 Graphics by Gerald Jarnig 2024

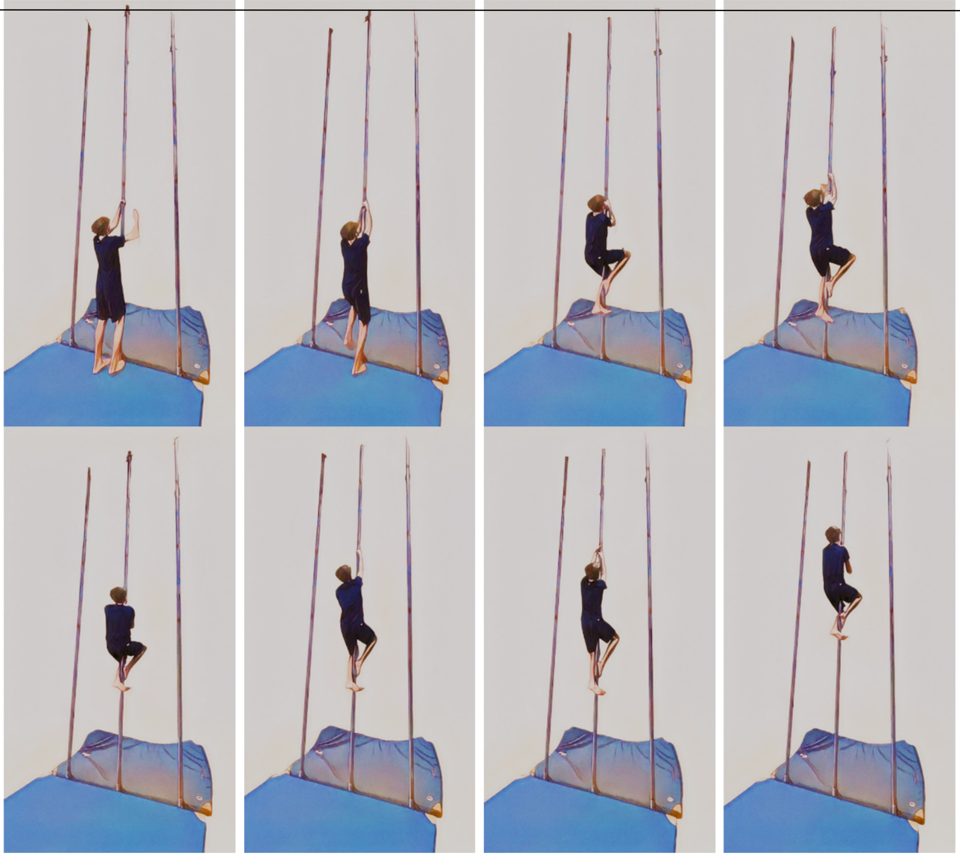

Climbing technique Austrian Pole Climbing Test - Leg holding technique variant 2 Graphics by Gerald Jarnig 2024

Test procedure

A test person should complete as many climbing attempts as possible on the climbing pole within a time span of 2 minutes, using the correct climbing technique. The test administrator provides the test person with continuous information during a climbing attempt regarding the climbing height/climbing achievement. Each height climbed over with the chin is recorded and communicated to the test person verbally. The break between the individual climbing attempts within the 2 minutes is chosen by the participants themselves, depending on their state of physical fatigue. The participants are asked to keep breaks as short as possible and to complete the maximum possible total climbing height. There is no time limit for the last climbing attempt started within the 2 minutes, this may be carried out until physical exhaustion and the maximum norm mark reached is included in the overall assessment. The participant must start climbing from an upright position, an active jump upwards onto the pole is an incorrect climbing technique, the climbing attempt must be stopped immediately in such a case and the next climbing attempt must be started with the correct climbing technique.

Execution errors / sources of error

The most typical execution error in the climbing technique is the climb climbing. Here, the entire body is positioned in the same alignment to the climbing pole. The soles of both legs are pressed against the poles and the arms are held against the poles with a double grip. In this position, the part of the body furthest away from the pole is the buttocks, the arms are stretched and the upper body and torso are away from the pole. There is an acute risk of injury, as an uncontrolled release of the arms/one arm can result in a fall backwards. If the participant uses this technique, the test administrator must stop the climbing attempt immediately. The test will be continued with the correct climbing technique as soon as possible.

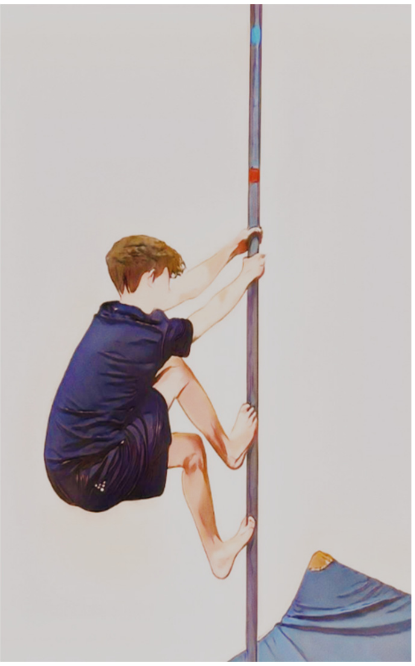

Execution error - climb climbing - Graphics by Gerald Jarnig 2024

Test duration and end of test

Test duration
The maximum test duration is open, the last climbing attempt must be started within a 2-minute time period.
End of test
The pole climbing test ends after 2 minutes; a climbing attempt carried out actively at this point can be completed without time pressure.

Test instruction

The test administrator gives the participants instructions as uniformly as possible. There are 3 different test instructions:
Before carrying out the test:
General information: „Please go to the climbing station, you have 2 minutes to start as many climbing attempts as possible. Every norm mark you climb over with your chin will be documented by me. As soon as you have climbed over the highest norm mark, climb down

safely, stand with both legs on the gym mat and start the next climbing attempt as soon as possible. If your muscle strength is completely exhausted, rest as short as possible and start climbing again as soon as possible.“

During the test execution:
The test administrator provides information about the remaining test time every 30 seconds and 10 seconds before the end of the total test time.

> „You have 90 (60/30/10) seconds left!“

The test administrator verbally informs about each norm mark climbed over.

> „Height 2.0 (2.5/3.0/3.5/4.0) reached!“

At the end of the 2-minute testing time:
The test administrator informs about the time period in which climbing attempts may be started.

> „Please complete the current climbing attempt in a calm manner, the time in which you are allowed to start climbing attempts has now ended!“

Test documentation / Test administration

Recording of measured values

The assessment sheet, a recording device and a stopwatch are required to record the measured values.
The test administrator should go to the test administrator station and keep an eye on the assessment sheet, the stopwatch and the climbing station at the same time. The assessment sheet is used to record which norm marks were climbed over in the individual climbing attempts.

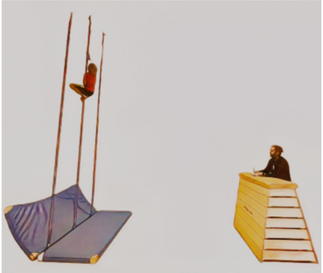

Recording of measured values - Graphics by Gerald Jarnig 2024

Overclimbed norm marks

If a participant succeeds in positioning the chin above a norm mark using the correct climbing technique, this norm mark is considered to have been climbed over.

Successful overclimbing:

Norm mark clearly climbed over

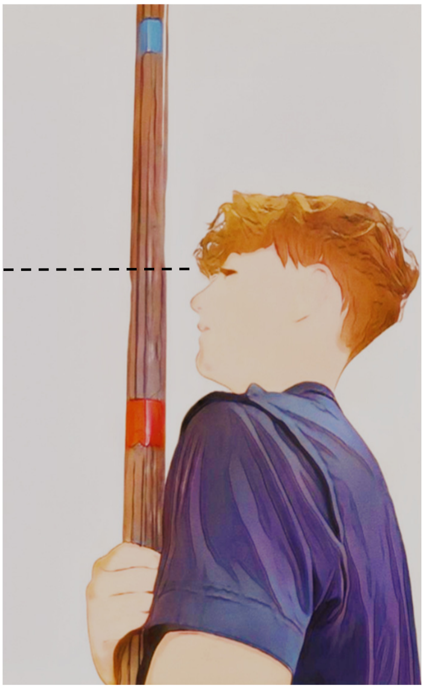

Measured standard: Norm mark successfully climbed over - Graphics by Gerald Jarnig 2024

Test assessment sheet

The test assessment sheet is used to document the test achievement.
The test assessment sheet is filled out by the test administrator in the following order:

General test data:	① Date of testing<br>② Test group<br>③ Test administrator<br>④ Thickness of the safety mat
Personal test data:	⑤ Code of the participant<br>⑥ Grip height
Test achievements:	⑦ The test administrator marks the individual norm marks (in meters) which are successfully climbed over in continuous climbing attempts. ⑧ There is one assessment area for each climbing attempt. In total there are eighteen assessment areas.
Test result:	⑨ The total climbing height (in m) of max. 6 successful climbing attempts is calculated. ⑩ At the right end of the assessment sheet, in the scoring area, the total climbing height in meters (from the results of Ⓐ Ⓑ Ⓒ) is calculated for each participant and the number of climbing attempts and the grip height are documented.

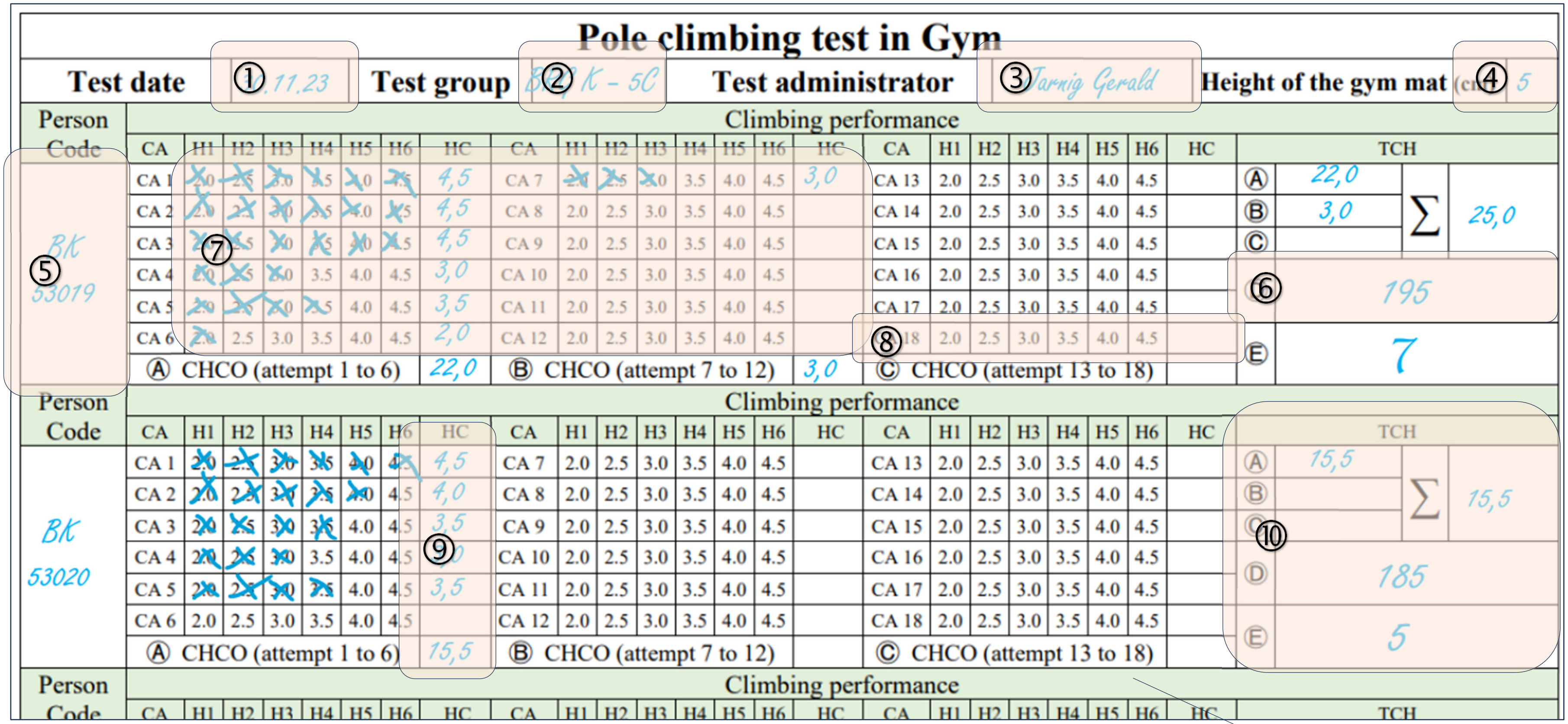

Pole climbing test in Gym

Test date	[illegible].11.23	Test group	[illegible]K - 5C	Test administrator	Jarnig Gerald	Height of the gym mat (cm)	5

Person Code	CA	H1	H2	H3	H4	H5	H6	HC	CA	H1	H2	H3	H4	H5	H6	HC	CA	H1	H2	H3	H4	H5	H6	HC	TCH	
BK 53019	CA 1	2.0	2.5	3.0	3.5	4.0	4.5	4,5	CA 7	2.0	2.5	3.0	3.5	4.0	4.5	3,0	CA 13	2.0	2.5	3.0	3.5	4.0	4.5		Ⓐ 22,0	Σ 25,0
	CA 2	2.0	2.5	3.0	3.5	4.0	4.5	4,5	CA 8	2.0	2.5	3.0	3.5	4.0	4.5		CA 14	2.0	2.5	3.0	3.5	4.0	4.5		Ⓑ 3,0	
	CA 3	2.0	2.5	3.0	3.5	4.0	4.5	4,5	CA 9	2.0	2.5	3.0	3.5	4.0	4.5		CA 15	2.0	2.5	3.0	3.5	4.0	4.5		Ⓒ	
	CA 4	2.0	2.5	3.0	3.5	4.0	4.5	3,0	CA 10	2.0	2.5	3.0	3.5	4.0	4.5		CA 16	2.0	2.5	3.0	3.5	4.0	4.5		Ⓓ 195	
	CA 5	2.0	2.5	3.0	3.5	4.0	4.5	3,5	CA 11	2.0	2.5	3.0	3.5	4.0	4.5		CA 17	2.0	2.5	3.0	3.5	4.0	4.5			
	CA 6	2.0	2.5	3.0	3.5	4.0	4.5	2,0	CA 12	2.0	2.5	3.0	3.5	4.0	4.5		CA 18	2.0	2.5	3.0	3.5	4.0	4.5		Ⓔ 7	
	Ⓐ CHCO (attempt 1 to 6)							22,0	Ⓑ CHCO (attempt 7 to 12)							3,0	Ⓒ CHCO (attempt 13 to 18)									
BK 53020	CA 1	2.0	2.5	3.0	3.5	4.0	4.5	4,5	CA 7	2.0	2.5	3.0	3.5	4.0	4.5		CA 13	2.0	2.5	3.0	3.5	4.0	4.5		Ⓐ 15,5	Σ 15,5
	CA 2	2.0	2.5	3.0	3.5	4.0	4.5	4,0	CA 8	2.0	2.5	3.0	3.5	4.0	4.5		CA 14	2.0	2.5	3.0	3.5	4.0	4.5		Ⓑ	
	CA 3	2.0	2.5	3.0	3.5	4.0	4.5	3,5	CA 9	2.0	2.5	3.0	3.5	4.0	4.5		CA 15	2.0	2.5	3.0	3.5	4.0	4.5		Ⓒ	
	CA 4	2.0	2.5	3.0	3.5	4.0	4.5	[illegible]	CA 10	2.0	2.5	3.0	3.5	4.0	4.5		CA 16	2.0	2.5	3.0	3.5	4.0	4.5		Ⓓ 185	
	CA 5	2.0	2.5	3.0	3.5	4.0	4.5	3,5	CA 11	2.0	2.5	3.0	3.5	4.0	4.5		CA 17	2.0	2.5	3.0	3.5	4.0	4.5			
	CA 6	2.0	2.5	3.0	3.5	4.0	4.5		CA 12	2.0	2.5	3.0	3.5	4.0	4.5		CA 18	2.0	2.5	3.0	3.5	4.0	4.5		Ⓔ 5	
	Ⓐ CHCO (attempt 1 to 6)							15,5	Ⓑ CHCO (attempt 7 to 12)								Ⓒ CHCO (attempt 13 to 18)									

Callouts: ① ② ③ ④ ⑤ ⑥ ⑦ ⑧ ⑨ ⑩

Instructions for the test assessment sheet - Austrian Pole Climbing Test - Graphics by Gerald Jarnig 2024

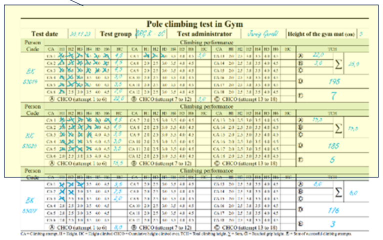

Pole climbing test in Gym

Test date		Test group		Test administrator		Height of the gym mat (cm)	

Person Code	Climbing performance																										
	CA	H1	H2	H3	H4	H5	H6	HC	CA	H1	H2	H3	H4	H5	H6	HC	CA	H1	H2	H3	H4	H5	H6	HC	TCH		
	CA 1	2.0	2.5	3.0	3.5	4.0	4.5		CA 7	2.0	2.5	3.0	3.5	4.0	4.5		CA 13	2.0	2.5	3.0	3.5	4.0	4.5		Ⓐ		∑
	CA 2	2.0	2.5	3.0	3.5	4.0	4.5		CA 8	2.0	2.5	3.0	3.5	4.0	4.5		CA 14	2.0	2.5	3.0	3.5	4.0	4.5		Ⓑ		
	CA 3	2.0	2.5	3.0	3.5	4.0	4.5		CA 9	2.0	2.5	3.0	3.5	4.0	4.5		CA 15	2.0	2.5	3.0	3.5	4.0	4.5		Ⓒ		
	CA 4	2.0	2.5	3.0	3.5	4.0	4.5		CA 10	2.0	2.5	3.0	3.5	4.0	4.5		CA 16	2.0	2.5	3.0	3.5	4.0	4.5		Ⓓ		
	CA 5	2.0	2.5	3.0	3.5	4.0	4.5		CA 11	2.0	2.5	3.0	3.5	4.0	4.5		CA 17	2.0	2.5	3.0	3.5	4.0	4.5				
	CA 6	2.0	2.5	3.0	3.5	4.0	4.5		CA 12	2.0	2.5	3.0	3.5	4.0	4.5		CA 18	2.0	2.5	3.0	3.5	4.0	4.5		Ⓔ		
	Ⓐ CHCO (attempt 1 to 6)								Ⓑ CHCO (attempt 7 to 12)								Ⓒ CHCO (attempt 13 to 18)										
Person Code	Climbing performance																										
	CA	H1	H2	H3	H4	H5	H6	HC	CA	H1	H2	H3	H4	H5	H6	HC	CA	H1	H2	H3	H4	H5	H6	HC	TCH		
	CA 1	2.0	2.5	3.0	3.5	4.0	4.5		CA 7	2.0	2.5	3.0	3.5	4.0	4.5		CA 13	2.0	2.5	3.0	3.5	4.0	4.5		Ⓐ		∑
	CA 2	2.0	2.5	3.0	3.5	4.0	4.5		CA 8	2.0	2.5	3.0	3.5	4.0	4.5		CA 14	2.0	2.5	3.0	3.5	4.0	4.5		Ⓑ		
	CA 3	2.0	2.5	3.0	3.5	4.0	4.5		CA 9	2.0	2.5	3.0	3.5	4.0	4.5		CA 15	2.0	2.5	3.0	3.5	4.0	4.5		Ⓒ		
	CA 4	2.0	2.5	3.0	3.5	4.0	4.5		CA 10	2.0	2.5	3.0	3.5	4.0	4.5		CA 16	2.0	2.5	3.0	3.5	4.0	4.5		Ⓓ		
	CA 5	2.0	2.5	3.0	3.5	4.0	4.5		CA 11	2.0	2.5	3.0	3.5	4.0	4.5		CA 17	2.0	2.5	3.0	3.5	4.0	4.5				
	CA 6	2.0	2.5	3.0	3.5	4.0	4.5		CA 12	2.0	2.5	3.0	3.5	4.0	4.5		CA 18	2.0	2.5	3.0	3.5	4.0	4.5		Ⓔ		
	Ⓐ CHCO (attempt 1 to 6)								Ⓑ CHCO (attempt 7 to 12)								Ⓒ CHCO (attempt 13 to 18)										
Person Code	Climbing performance																										
	CA	H1	H2	H3	H4	H5	H6	HC	CA	H1	H2	H3	H4	H5	H6	HC	CA	H1	H2	H3	H4	H5	H6	HC	TCH		
	CA 1	2.0	2.5	3.0	3.5	4.0	4.5		CA 7	2.0	2.5	3.0	3.5	4.0	4.5		CA 13	2.0	2.5	3.0	3.5	4.0	4.5		Ⓐ		∑
	CA 2	2.0	2.5	3.0	3.5	4.0	4.5		CA 8	2.0	2.5	3.0	3.5	4.0	4.5		CA 14	2.0	2.5	3.0	3.5	4.0	4.5		Ⓑ		
	CA 3	2.0	2.5	3.0	3.5	4.0	4.5		CA 9	2.0	2.5	3.0	3.5	4.0	4.5		CA 15	2.0	2.5	3.0	3.5	4.0	4.5		Ⓒ		
	CA 4	2.0	2.5	3.0	3.5	4.0	4.5		CA 10	2.0	2.5	3.0	3.5	4.0	4.5		CA 16	2.0	2.5	3.0	3.5	4.0	4.5		Ⓓ		
	CA 5	2.0	2.5	3.0	3.5	4.0	4.5		CA 11	2.0	2.5	3.0	3.5	4.0	4.5		CA 17	2.0	2.5	3.0	3.5	4.0	4.5				
	CA 6	2.0	2.5	3.0	3.5	4.0	4.5		CA 12	2.0	2.5	3.0	3.5	4.0	4.5		CA 18	2.0	2.5	3.0	3.5	4.0	4.5		Ⓔ		
	Ⓐ CHCO (attempt 1 to 6)								Ⓑ CHCO (attempt 7 to 12)								Ⓒ CHCO (attempt 13 to 18)										

CA = Climbing attempt, H = Height, HC = Height climbed CHCO = Cumulative height climbed over, TCH = Total climbing height, ∑ = Sum, Ⓓ = Reached grip height, Ⓔ = Sum of successful climbing attempts.

Test evaluation

The achievements of the successfully completed climbing attempts are summarized in the scoring area on the test assessment sheet.

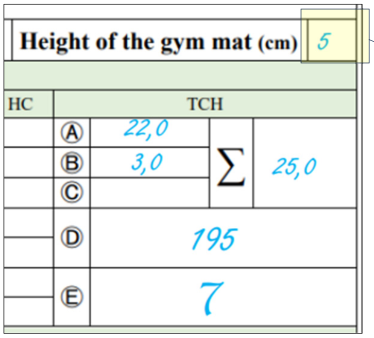

Height of the gym mat (cm)		5	
HC	TCH		
	Ⓐ	22,0	∑ 25,0
	Ⓑ	3,0	
	Ⓒ		
	Ⓓ	195	
	Ⓔ	7	

Ⓕ

Scoring area on the assessment sheet - Austrian Pole Climbing Test - © JG, 2024

Ⓐ = Sum of the heights climbed over from climbing attempt 1 to 6
Ⓑ = Sum of the heights climbed over from climbing attempt 7 to 12
Ⓒ = Sum of the heights climbed over from climbing attempt 13 to 18
Ⓓ = Grip height (in cm)
Ⓔ = Number of successful climbing attempts
Ⓕ = Thickness of the safety mat (in cm)
∑ = Total climbing height (in m)

The individual total climbing height is calculated using the data in the scoring area.

Calculation of the individual total climbing height:
Formula:

$$Individual\ total\ climbing\ height_{(in\ Meter)} = \frac{\sum \times 100 - (D + F) \times E}{100}$$

Note with Ⓕ = When the thickness of the safety mat is 20 cm or more, a compression of 5 cm of the mat caused by the weight of the participant is taken into account for the calculation. The formula is then modified as follows:

$$Individual\ total\ climbing\ height_{(in\ Meter)} = \frac{\sum \times 100 - (D + F) \times E}{100} + E \times F$$

Assessment of performance

Two different assessment models are available for performance assessment, which enable a general classification of performance into 3 performance categories (low, average and high climbing performance) and a more detailed classification of performance into 9 performance levels.

Categories of the Austrian Pole Climbing Test:
The reference data was generated in a pilot study during the development of the field test and classified using a nine-point rating system.
The reference values are for children, adolescents and young adults of school age and above who demonstrate achievement in the pole climbing test.
A positive achievement is defined as climbing over a norm mark.

**Variable**	**Gender**	**Age group**	**Categories of performance according to the Austrian Pole Climbing Test**								
			1	2	3	4	5	6	7	8	9
**Reference values [m]**	**Male**	≤ 7	≤ 0.00	0.01 - 0.40	0.41 - 1.37	1.38 - 2.35	2.36 - 3.32	3.33 - 4.29	4.30 - 5.26	5.27 - 6.23	> 6.23
		8 to 9	≤ 0.00	0.01 - 0.43	0.44 - 2.02	2.03 - 3.60	3.61 - 5.19	5.20 - 6.78	6.79 - 8.37	8.38 - 9.95	> 9.95
		10 to 11	≤ 0.00	0.01 - 0.68	0.69 - 2.59	2.60 - 4.51	4.52 - 6.42	6.43 - 8.34	8.35 - 10.26	10.27 - 12.17	> 12.17
		12 to 13	≤ 0.00	0.01 - 1.95	1.96 - 4.15	4.16 - 6.36	6.37 - 8.56	8.57 - 10.77	10.78 - 12.97	12.98 - 15.18	> 15.18
		14 to 15	≤ 0.00	0.01 - 2.96	2.97 - 5.32	5.33 - 7.68	7.69 - 10.03	10.04 - 12.39	12.40 - 14.75	14.76 - 17.11	> 17.11
		16 to 17	≤ 0.00	0.01 - 2.77	2.78 - 5.58	5.59 - 8.39	8.40 - 11.20	11.21 - 14.00	14.01 - 16.81	16.82 - 19.62	> 19.62
		18 to 19	≤ 0.00	0.01 - 5.33	5.34 - 7.02	7.03 - 8.71	8.72 - 10.40	10.41 - 12.09	12.10 - 13.77	13.78 - 15.46	> 15.46
	**Female**	≤ 7	≤ 0.00	0.01 - 0.21	0.22 - 1.00	1.01 - 1.78	1.79 - 2.57	2.58 - 3.35	3.36 - 4.14	4.15 - 4.93	> 4.93
		8 to 9	≤ 0.00 (Category 1)		0.01 - 1.53	1.54 - 4.00	4.01 - 6.48	6.49 - 8.95	8.96 - 11.42	11.43 - 13.90	> 13.90
		10 to 11	≤ 0.00 (Category 1)		0.01 - 2.00	2.01 -4.21	4.22 - 6.42	6.43 - 8.63	8.64 - 10.83	10.84 - 13.04	> 13.04
		12 to 13	≤ 0.00	0.01 - 0.17	0.18 - 2.44	2.45 - 4.72	4.73 - 6.99	7.00 - 9.27	9.28 - 11.54	11.55 - 13.82	> 13.82
		14 to 15	≤ 0.00	0.01 - 0.16	0.17 - 2.07	2.08 - 3.98	3.99 - 5.89	5.90 - 7.80	7.81 - 9.71	9.72 - 11.62	> 11.62
		16 to 17	≤ 0.00	0.01 - 0.16	0.17 - 1.86	1.87 - 3.56	3.57 - 5.26	5.27 - 6.97	6.98 - 8.67	8.68 - 10.37	> 10.37
		18 to 19	≤ 0.00	0.01 - 0.49	0.50 - 1.64	1.65 - 2.78	2.79 - 3.93	3.94 - 5.08	5.09 - 6.22	6.23 - 7.37	> 7.37

**Categories of classification**					
Low climbing performance		Average climbing performance		High climbing performance	
1	No climbing performance	4	Below-average climbing performance	7	Good climbing performance
2	poor climbing performance	5	Average climbing performance	8	Very good climbing performance
3	Weak climbing performance	6	Above-average climbing performance	9	Excellent climbing performance

m=meter.

Potential sources of error

- Jumping up onto the climbing pole at the start of a climbing attempt.
- Incorrect climbing technique.
- Confusing the assessment area.
- Overlooking the climbing over a norm mark.
- The test is carried out with sports shoes.
- A test subject is interrupted by another participant during the test.

Further information

The test is carried out in the sports hall on standardized, smooth, round climbing poles fixed at the top and bottom in the use position. Depending on the design of the climbing station, up to a maximum of 4 participants (with 2 test administrators) can be assessed at the same time.

Supplementary material

Tables and Figures

Table S1. Application in Austria of pole climbing in selection procedures

Institutions in Austria		Field of application	Sex	Description
Ministry	Federal Ministry of Education, Science and Research	School sports competition - Versatility competition (5th & 6th grades)	M/F	Pole climb - max. 3 pole heights, time limit max. 2 min.
University	University Innsbruck	Admission test for sports studies	F	Climb up the pole as quickly as possible
	Private University College of Education of the Diocese of Linz	Admission test for sports teaching	M/F	Climb along seven poles - horizontally
	University College of Teacher Education Vienna/Krems			Pole climb - max. 2 pole heights, time limit max. 2 min.
Secondary School & High School	BG / BRG Porcia Spittal/Drau	Sports motor admission test	M/F	Pole climbing - no detailed information
	BRG/BORG Klagenfurt			Austrian Pole Climbing Test - (Location of the test development)
	BORG Deutsch-Wagram			Climb up a pole (approx. 1.0 m) and then climb horizontally to another pole
	Christan Doppler Gymnasium			
	Göthe Gymnasium Wien			
	BRG Wels/Wallerstraße			Climb up the pole as quickly as possible
	Bundesgymnasium Zehnergasse Wiener Neustadt			
	Bundesrealgymnasium Kremszeile			
	Georg von Peuerbach Gymnasium			
	RG Sport Musik Salzburg			
	Sportgymnasium Hartberg			Pole climb - height max. 1.5 (M) / 1.0 (F) pole heights, without time limit
	BRG/BORG Kirchdorf			Pole climb - height max. 2 pole heights, without time limit
	Bundesgymnasium Wiener Neustadt			
	Sportgymnasium Maria Enzersdorf			
	Sportgymnasium St.Pölten			
	Sportrealgymnasium Leibnitz			Pole climb - height max. 4 pole heights, without time limit
Secondary School	SMS Donaustadt	Sports motor admission test	M/F	Climb up the pole as quickly as possible
	SMS Gmünd			
	SMS Hohenems-Markt			
	SMS Imst			
	SMS Scheibbs			
	SMS St.Pölten			
	SMS Satteins			Pole climb - height max. 2 pole heights, without time limit / 1 Point per climbed mark
	SMS Sandl			Pole climb - height max. 3 Meters
	SMS Mondsee			Pole climb - height max. 3 pole heights, without time limit
	SMS Gars am Kamp			Pole climb - height max. 4 pole heights, without time limit
	SMS Laa an der Thaya			
	SMS Wals-Siezenheim			
	SMS Wölbling			
	NMS Laakirchen			Pole climbing - no detailed information
	NMS Neusiedl			
	SMS Bad Vöslau			
	SMS Egger Lienz			
	SMS Graz			

	SMS Hadersdorf			
	SMS Hohenems			
	SMS Niederwaldkirchen			
	SMS St.Valentin			
	SMS Tulln			

Table S2. Classification of the Austrian Pole Climbing Test

z value	APCT Category		
< -1.75	1	Low c.p.	No c.p.
-1.74 bis -1.25	2		poor c.p.
-1.24 bis -0.75	3		Weak c.p.
-0.74 bis -0.25	4	Average c.p.	Below-average c.p.
-0.24 bis 0.25	5		Average c.p.
0.26 bis 0.75	6		Above-average c.p.
0.76 bis 1.25	7	High c.p.	Good c.p.
1.26 bis 1.75	8		Very good c.p.
≥ 1.75	9		Excellent c.p.

APCT = Austrian Pole Climbing Test, c.p. = climbing performance;

Table S3. Differences between participants with and without achievements at the Austrian Pole Climbing Test

Variable	Achievements at APCT [677]	No - Achievements at APCT [278]	Achievements vs. No - Achievements		
			t	*P* value	p-lvl
**Age, years**	13.4 (3.3)	12.8 (3.6)	2.336	0.020	*
**Weight, kg**	50.5 (17.3)	53.9 (20.2)	2.654	0.008	**
**Height, cm**	159.1 (18.3)	154.6 (17.5)	3.430	<.001	***
**BMI, kg/m2**	19.2 (3.2)	21.7 (5.0)	7.683	<.001	***
**EQUI BMIAUT, mean (SD)**	21.7 (2.5)	24.6 (4.9)	9.275	<.001	***
**Austria weight classification**					
Underweight	50 (7.4)	17 (6.1)	p-lvl = ***<br>(see Tab. S4)		
Normal weight	564 (83.3)	147 (52.9)			
Overweight	59 (8.7)	74 (26.6)			
Obesity	4 (0.6)	31 (11.2)			
Morbid obesity	0 (0.0)	9 (3.2)			

N = Study population, Data are No. (%) or mean (SD).; No. = number, % = Percent, SD = standard deviation, kg = kilogram, cm = centimeter, BMI = body mass index, kg/m2 = kilogram / meter squared, EQUI BMI_{AUT} = equivalent BMI based on Austrian reference centile curves passing through adult BMI values [1], APCT = Austrian Pole Climbing Test, *t* = *t*-test; p-lvl (P Value level): * = P < 0.05, ** = P < 0.01, *** = P < 0.001.

Table S4. Mann-Whitney U-test used to assess differences in classification groups between participants with and without achievements at the Austrian Pole Climbing Test

Variable	Achievements vs. No - Achievements					
	U	Z	P value	p-lvl	r	E.s.
**AUT weight classification**	64340	10.056	<.001	***	0.33	M

U = Test statistic, Z = z-statistic, p-lvl (P Value level): * = P < 0.05, ** = P < 0.01, *** = P < 0.001, r = Pearson correlation coefficient, E.s. = effect size, M = moderate correlation; AUT = Austrian.

Table S5. Differences between participants with and without APCT achievements in age and gender-specific subgroups

Age group	Group	N	Achievements at APCT	No - Achievements at APCT	X²	P value	p-lvl
**All**	**Boys**	**552**	456 (82.6)	96 (17.4)	87.049	<.001	***
	**Girls**	**403**	221 (54.8)	182 (45.2)			
**≤ 7**	**Boys**	**47**	27 (57.4)	20 (42.6)	0.148	0.70	
	**Girls**	**39**	24 (61.5)	15 (38.5)			
**8 to 9**	**Boys**	**69**	50 (72.5)	19 (27.5)	3.809	0.05	
	**Girls**	**49**	27 (55.1)	22 (44.9)			
**10 to 11**	**Boys**	**78**	63 (80.8)	16 (19.2)	4.018	0.045	*
	**Girls**	**68**	45 (66.2)	23 (33.8)			
**12 to 13**	**Boys**	**95**	70 (73.7)	25 (26.3)	6.165	0.013	*
	**Girls**	**54**	29 (53.7)	25 (46.3)			
**14 to 15**	**Boys**	**148**	139 (93.9)	9 (6.1)	63.275	<.001	***
	**Girls**	**74**	35 (47.3)	39 (52.7)			
**16 to 17**	**Boys**	**79**	72 (91.1)	7 (8.9)	25.905	<.001	***
	**Girls**	**86**	48 (55.8)	38 (44.2)			
**18 to 19**	**Boys**	**36**	35 (97.2)	1 (2.8)	27.195	<.001	***
	**Girls**	**33**	13 (39.4)	20 (60.6)			

N = Study population, Data are No. (%) or mean (SD).; No. = number, % = Percent, SD = standard deviation, X^2 = Chi-Square Test value, , P-value = levels (p-lvl): *= $p < 0.05$; ** = $p < 0.01$; and *** = $p < 0.001$, APCT = Austrian Pole Climbing Test.

Table S6. Overall sample characteristics

Variable				All	Boys	Girls
N				677	456	221
Age, years				13.4 (3.3)	13.6 (3.2)	12.9 (3.5)
Anthropometric data		Weight, kg		50.5 (17.3)	53.3 (17.9)	44.7 (14.2)
		Height, cm		159.1 (18.3)	162.5 (18.6)	151.9 (15.5)
		BMI, kg/m2		19.2 (3.2)	19.4 (3.1)	18.8 (3.4)
		EQUI BMI_{AUT}, mean (SD)		21.7 (2.5)	22.0 (2.3)	22.7 (4.2)
		AUT weight classification, No. (%)				
		Underweight		50 (7.4)	18 (3.9)	32 (14.5)
		Normal weight		564 (83.3)	395 (86.6)	169 (76.5)
		Overweight		59 (8.7)	41 (9.0)	18 (8.1)
		Obesity		4 (0.6)	2 (0.4)	2 (0.9)
		Morbid obesity		0 (0.0)	0 (0.0)	0 (0.0)
Sport data	APCT, m			6.59 (4.77)	7.53 (4.84)	4.66 (3.98)
	Test of Reliability and Objectivity	N		92	68	24
		Age, years		12.8 (1.2)	12.7 (1.2)	12.8 (1.1)
		EQUI BMI_{AUT}, mean (SD)		21.3 (2.1)	21.6 (2.0)	20.5 (2.2)
		APCT R1 T1, m		6.29 (3.71)	6.07 (3.73)	6.90 (3.68)
		APCT R1 T2, m		6.49 (3.64)	6.24 (3.60)	7.21 (3.75)
		APCT R2 T1, m		6.29 (3.73)	6.08 (3.75)	6.90 (3.67)
	Test of Validity	N		89	66	23
		Age, years		12.8 (1.2)	12.7 (1.2)	12.8 (1.1)
		MHF - R, kg/kg-bw		0.75 (0.10)	0.76 (0.10)	0.72 (0.11)
		MHF - L, kg/kg-bw		0.71 (0.11)	0.72 (0.11)	0.67 (0.10)
		PU - OG, No.		4.4 (3.5)	4.6 (3.7)	3.7 (2.9)
		PU - UG, No.		5.9 (4.6)	6.2 (4.9)	5.0 (3.6)
		APCT, m		6.28 (3.71)	6.04 (3.72)	6.90 (3.76)
		N		289	180	109
		Age, years		10.8 (2.3)	11.0 (2.3)	10.4 (2.2)
		Push-ups, No.		15.0 (3.6)	15.3 (3.9)	14.5 (3.2)
		APCT, m		5.32 (4.20)	5.67 (4.12)	4.75 (4.29)
		N		617	412	205
		Age, years		13.2 (3.3)	13.4 (3.2)	12.9 (3.6)
		SLJ, cm		175.0 (38.3)	187.6 (36.1)	150.0 (28.9)
		APCT, rm		6.45 (4.65)	7.30 (4.69)	4.75 (4.05)
	Competitiveness with established pole climbing field test	N		85	61	24
		Age, years		12.2 (1.2)	12.2 (1.2)	12.3 (1.4)
		RCH, m		2.01 (0.14)	2.01 (0.14)	2.01 (0.15)
		R RCH, m		0.75 (1.68 - 2.43)	0.61 (1.68 - 2.29)	0.68 (1.75 - 2.43)
		APCT, m		8.09 (4.45)	7.79 (4.67)	8.86 (3.79)
		Performance categories APCT	No C.P.	2 (2.4)	2 (3.3)	0 (0.0)
			poor C.P.	1 (1.2)	1 (1.6)	0 (0.0)
			Weak C.P.	7 (8.2)	7 (11.5)	0 (0.0)
			Below-average C.P.	12 (14.1)	8 (13.1)	4 (16.7)
			Average C.P.	21 (24.7)	16 (26.2)	5 (20.8)
			Above-average C.P.	12 (14.1)	8 (13.1)	4 (16.7)
			Good C.P.	15 (17.6)	9 (14.8)	6 (25.0)
			Very good C.P.	5 (5.9)	4 (6.6)	1 (4.2)
			Excellent C.P.	10 (11.8)	6 (9.8)	4 (16.7)
			SPC, sec	7.7 (2.9)	7.8 (3.1)	7.4 (2.5)
		Performance categories SPC	not enough C.P.	3 (3.5)	2 (3.3)	0 (0.0)
			sufficient C.P.	3 (3.5)	3 (4.9)	1 (4.2)
			satisfactory C.P.	25 (29.4)	24 (39.3)	1 (4.2)
			good C.P.	16 (18.8)	13 (21.3)	3 (12.5)
			very good C.P.	38 (44.7)	19 (31.1)	19 (79.2)

N = Study population, Data are No. (%) or mean (SD).; No. = number, % = Percent, SD = standard deviation, kg = kilogram, cm = centimeter, BMI = body mass index, , kg/m2 = kilogram / meter squared, kg/kg-bw = Kilogram per kilogram of body weight, EQUI BMI_{AUT}

= equivalent BMI based on Austrian reference centile curves passing through adult BMI values, APCT = Austrian Pole Climbing Test, T = Measurement time point, R = Rater, MHF – R = maximum Hand force – right Hand, MHF – L = maximum Hand force – left Hand, PU – OG = Pull-ups - over grip, PU – UG = Pull-ups - under grip, SPC = Speed pole climbing, RCH = Real climbing height, R – RCH = Range - Real climbing height, C.P. = Climbing Performance.

Table S7. Gender and age-specific mean values of participants with achievements at the Austrian Pole Climbing Tests

Age group	Gender	Mean values, (m) – Austrian Pole Climbing Test		t	*P* value	p-lvl
		Mean	SD			
≤ 7	♂ (N=27)	2.83	1.94	1.316	.19	
	♀ (N=24)	2.18	1.57			
8 to 9	♂ (N=50)	4.40	3.18	-.798	.43	
	♀ (N=27)	5.24	4.95			
10 to 11	♂ (N=63)	5.47	3.83	.192	.85	
	♀ (N=45)	5.31	4.42			
12 to 13	♂ (N=70)	7.46	4.41	1.632	.11	
	♀ (N=29)	5.85	4.55			
14 to 15	♂ (N=139)	8.85	4.72	4.548	<.001	***
	♀ (N=35)	4.94	3.82			
16 to 17	♂ (N=72)	9.79	5.62	6.525	<.001	***
	♀ (N=48)	4.41	3.40			
18 to 19	♂ (N=35)	9.55	3.38	6.095	<.001	***
	♀ (N=13)	3.36	2.29			

N = Study population, Data are No. or mean (SD).; No. = number, SD = standard deviation, *t* = *t*-test; p-lvl (P Value level): * = P < 0.05, ** = P < 0.01, *** = P < 0.001.

Table S8. Multiple comparisons between the gender-specific age categories for differences in achievement at APCT using the Games-Howell post-hoc test.

Sex	Age groups		Mean Difference (m)	95% Confidence Interval (m)		Std. Error	*P* value	p-lvl
				Lower Bound	Upper Bound			
Boys (N=456)	AC 1	AC 2	-1.57	-3.34	0.21	0.58444	.12	
		AC 3	-2.63	-4.48	-0.79	0.61070	.001	**
		AC 4	-4.63	-6.57	-2.68	0.64633	<.001	***
		AC 5	-6.02	-7.67	-4.37	0.54771	<.001	***
		AC 6	-6.96	-9.25	-4.67	0.76032	<.001	***
		AC 7	-6.72	-8.81	-4.63	0.68237	<.001	***
	AC 2	AC 3	-1.07	-3.05	0.91	0.65926	.67	
		AC 4	-3.06	-5.14	-0.98	0.69239	<.001	***
		AC 5	-4.46	-6.26	-2.65	0.60137	<.001	***
		AC 6	-5.39	-7.79	-2.99	0.79984	<.001	***
		AC 7	-5.16	-7.36	-2.95	0.72614	<.001	***
	AC 3	AC 4	-1.99	-4.13	0.15	0.71470	.09	
		AC 5	-3.39	-5.26	-1.51	0.62693	<.001	***
		AC 6	-4.33	-6.78	-1.87	0.81923	<.001	***
		AC 7	-4.09	-6.35	-1.82	0.74745	<.001	***
	AC 4	AC 5	-1.40	-3.37	0.58	0.66169	.35	
		AC 6	-2.33	-4.87	0.20	0.84612	.09	
		AC 7	-2.10	-4.44	0.25	0.77683	.11	
	AC 5	AC 6	-0.94	-3.26	1.38	0.77341	.89	
		AC 7	-0.70	-2.81	1.42	0.69693	.95	
	AC 6	AC 7	0.24	-2.39	2.87	0.87396	>.99	
Girls (N=221)	AC 1	AC 2	-3.06	-6.22	0.10	1.00477	.06	
		AC 3	-3.14	-5.37	-0.90	0.73236	.001	**
		AC 4	-3.68	-6.50	-0.86	0.90380	.004	**
		AC 5	-2.76	-4.98	-0.54	0.72101	.006	**
		AC 6	-2.24	-4.02	-0.46	0.58678	.005	**
		AC 7	-1.18	-3.53	1.17	0.71241	.65	
	AC 2	AC 3	-0.08	-3.63	3.48	1.15760	>.99	
		AC 4	-0.62	-4.52	3.29	1.27300	>.99	
		AC 5	0.30	-3.24	3.84	1.15045	>.99	
		AC 6	0.82	-2.50	4.15	1.07144	.997	
		AC 7	1.88	-1.68	5.44	1.14508	.66	
	AC 3	AC 4	-0.54	-3.81	2.73	1.07113	>.99	
		AC 5	0.38	-2.42	3.17	0.92213	>.99	
		AC 6	0.90	-1.58	3.38	0.82145	.93	
		AC 7	1.96	-0.89	4.80	0.91542	.35	
	AC 4	AC 5	0.92	-2.34	4.17	1.06340	.98	
		AC 6	1.44	-1.57	4.45	0.97738	.76	
		AC 7	2.50	-0.79	5.78	1.05759	.24	
	AC 5	AC 6	0.52	-1.94	2.99	0.81134	>.99	
		AC 7	1.58	-1.25	4.41	0.90636	.59	
	AC 6	AC 7	1.06	-1.49	3.61	0.80371	.84	

N = Study population, m = meter, AC = Age category, AC 1 = ≤ 7 years, AC 2 = 8 to 9 years, AC 3 = 10 to 11 years, AC 4 = 12 to 13 years, AC 5 = 14 to 15 years, AC 6 = 16 to 17 years, AC 7 = 18 to 19 years, p-lvl (P Value level): * = P < 0.05, ** = P < 0.01, *** = P < 0.001.

Note: The Games-Howell post-hoc test was used because no homogeneity of variance could be found in a simple ANOVA.

Table S9. Performance thresholds for APCT defined by thresholds of traditional z-score standardization (-1.75, -1.25, -0.75, -0.25, 0.25, 0.75, 1.25 and 1.75) for the classification of a nine-point assessment (according to data from the own study population)

Gender	Age group	Grading of performance in APCT (in Meter) by z-value thresholds according to a nine-point classification							
		**-1.75**	**-1.25**	**-0.75**	**-0.25**	**0.25**	**0.75**	**1.25**	**1.75**
**Boys**	≤ 7	-0.57	0.40	1.37	2.35	3.32	4.29	5.26	6.23
	8 to 9	-1.16	0.43	2.02	3.60	5.19	6.78	8.37	9.95
	10 to 11	-1.24	0.68	2.59	4.51	6.42	8.34	10.26	12.17
	12 to 13	-0.26	1.95	4.15	6.36	8.56	10.77	12.97	15.18
	14 to 15	0.60	2.96	5.32	7.68	10.03	12.39	14.75	17.11
	16 to 17	-0.04	2.77	5.58	8.39	11.20	14.00	16.81	19.62
	18 to 19	3.65	5.33	7.02	8.71	10.40	12.09	13.77	15.46
**Girls**	≤ 7	-0.57	0.21	1.00	1.78	2.57	3.35	4.14	4.93
	8 to 9	-3.42	-0.95	1.53	4.00	6.48	8.95	11.42	13.90
	10 to 11	-2.41	-0.21	2.00	4.21	6.42	8.63	10.83	13.04
	12 to 13	-2.11	0.17	2.44	4.72	6.99	9.27	11.54	13.82
	14 to 15	-1.75	0.16	2.07	3.98	5.89	7.80	9.71	11.62
	16 to 17	-1.54	0.16	1.86	3.56	5.26	6.97	8.67	10.37
	18 to 19	-0.66	0.49	1.64	2.78	3.93	5.08	6.22	7.37

z-value = traditional z standardization; APCT = Austrian Pole Climbing Test.

Table S10. Descriptive statistics of the results for checking reliability and objectivity

Age group	Group	N	APCT R1 T1, m		Data to compare reliability		Data to compare objectivity	
					APCT R1 T2, m		APCT R2 T1, m	
			M	SD	M	SD	M	SD
10 to 11	All	19	6.04	3.86	6.04	3.79	6.01	3.91
	♂	14	5.77	3.64	5.77	3.47	5.73	3.71
	♀	5	6.80	4.81	6.79	4.94	6.80	4.81
12 to 13	All	56	6.18	3.63	6.55	3.51	6.20	3.65
	♂	42	6.13	3.82	6.38	3.64	6.16	3.84
	♀	14	6.32	3.12	7.05	3.17	6.32	3.10
14 to 15	All	17	6.92	3.96	6.80	4.09	6.92	3.96
	♂	12	6.22	3.79	6.27	3.89	6.22	3.79
	♀	5	8.60	4.27	8.06	4.74	8.60	4.27

N = Study population, Data are mean (SD).; SD = standard deviation, APCT = Austrian Pole Climbing Test, T = Measurement time point, m = meter, T1 = Baseline measurements made in autumn 2023, T2 = Measurement taken within 2 weeks of the baseline measurement, R1 = Rater 1, R2 = Rater 2, ♂ = Boys, ♀ = Girls.

Table S11. Reliability and objectivity of APCT performance in different age groups and as well separately for boys and girls

Test quality criteria	Age group	Group	N	ICC (2.1)	95% CI		Agreement
					Lower	Upper	
**Test of Reliability**	**10 to 11**	**All**	**19**	0.980	0.950	0.992	Excellent
		♂	**14**	0.971	0.911	0.991	Excellent
		♀	**5**	0.998	0.976	>.999	Excellent
	**12 to 13**	**All**	**56**	0.962	0.927	0.979	Excellent
		♂	**42**	0.971	0.946	0.984	Excellent
		♀	**14**	0.925	0.696	0.978	Moderate to Excellent
	**14 to 15**	**All**	**17**	0.977	0.939	0.992	Excellent
		♂	**12**	0.979	0.928	0.994	Excellent
		♀	**5**	0.975	0.823	0.997	Good to Excellent
**Test of objectivity**	**10 to 11**	**All**	**19**	0.999	0.997	0.999	Excellent
		♂	**14**	0.998	0.994	0.999	Excellent
		♀	**5**	>.999	>.999	>.999	Excellent
	**12 to 13**	**All**	**56**	0.999	0.998	0.999	Excellent
		♂	**42**	0.999	0.998	>.999	Excellent
		♀	**14**	0.998	0.994	0.999	Excellent
	**14 to 15**	**All**	**17**	>.999	>.999	>.999	Excellent
		♂	**12**	>.999	>.999	>.999	Excellent
		♀	**5**	>.999	>.999	>.999	Excellent

Explanation: To determine reliability Test [APCT R1 T1] – ReTest [APCT R1 T2]) and objectivity (Test rater 1 [APCT R1 T1] – Test rater 2 [APCT R2 T1]), the 95% CIs of the ICCs were interpreted as follows: 95% CI values below 0.5 were considered to indicate poor reliability, values between 0.5 and 0.75 were considered to indicate moderate reliability, values between 0.75 and 0.9 were considered to indicate good reliability, and values above 0.90 were considered to indicate excellent reliability. N = Study population, ICC = intraclass correlation, CI = confidence interval, APCT = Austrian Pole Climbing Test, ♂ = Boys, ♀ = Girls.

.

Table S12. Descriptive statistics of the results for checking validity

Variable			Sex	Age group																				
				≤ 7			8 to 9			10 to 11			12 to 13			14 to 15			16 to 17			18 to 19		
				N	M	SD	N	M	SD	N	M	SD	N	M	SD	N	M	SD	N	M	SD	N	M	SD
Upper body strength	Maximum hand force, pulling force endurance of the arms	MHF - R, kg/kg-bw	All	**ND**	ND	ND	**ND**	ND	ND	**17**	0.77	0.12	**55**	0.74	0.10	**17**	0.76	0.10	**ND**	ND	ND	**ND**	ND	ND
			♂	**ND**	ND	ND	**ND**	ND	ND	**13**	0.78	0.13	**41**	0.75	0.10	**12**	0.76	0.10	**ND**	ND	ND	**ND**	ND	ND
			♀	**ND**	ND	ND	**ND**	ND	ND	**4**	0.73	0.13	**14**	0.71	0.11	**5**	0.76	0.11	**ND**	ND	ND	**ND**	ND	ND
		MHF - L, kg/kg-bw	All	**ND**	ND	ND	**ND**	ND	ND	**17**	0.73	0.14	**55**	0.70	0.11	**17**	0.72	0.08	**ND**	ND	ND	**ND**	ND	ND
			♂	**ND**	ND	ND	**ND**	ND	ND	**13**	0.73	0.15	**41**	0.71	0.11	**12**	0.73	0.09	**ND**	ND	ND	**ND**	ND	ND
			♀	**ND**	ND	ND	**ND**	ND	ND	**4**	0.72	0.12	**14**	0.66	0.10	**5**	0.68	0.07	**ND**	ND	ND	**ND**	ND	ND
		PU - OG, No.	All	**ND**	ND	ND	**ND**	ND	ND	**17**	4.4	2.9	**55**	4.3	3.7	**17**	4.9	3.6	**ND**	ND	ND	**ND**	ND	ND
			♂	**ND**	ND	ND	**ND**	ND	ND	**13**	4.2	2.7	**41**	4.7	3.9	**12**	5.1	3.9	**ND**	ND	ND	**ND**	ND	ND
			♀	**ND**	ND	ND	**ND**	ND	ND	**4**	5.0	3.7	**14**	3.1	2.6	**5**	4.6	3.3	**ND**	ND	ND	**ND**	ND	ND
		PU - UG, No.	All	**ND**	ND	ND	**ND**	ND	ND	**17**	5.2	3.5	**55**	5.8	5.0	**17**	6.6	4.3	**ND**	ND	ND	**ND**	ND	ND
			♂	**ND**	ND	ND	**ND**	ND	ND	**13**	4.8	3.3	**41**	6.5	5.3	**12**	6.5	4.6	**ND**	ND	ND	**ND**	ND	ND
			♀	**ND**	ND	ND	**ND**	ND	ND	**4**	6.5	4.4	**14**	3.9	3.0	**5**	6.8	4.3	**ND**	ND	ND	**ND**	ND	ND
	APCT for validity check	APCT, m	All	**ND**	ND	ND	**ND**	ND	ND	**17**	6.75	3.42	**55**	5.93	3.75	**17**	6.92	3.96	**ND**	ND	ND	**ND**	ND	ND
			♂	**ND**	ND	ND	**ND**	ND	ND	**13**	6.21	3.37	**41**	5.94	3.89	**12**	6.22	3.79	**ND**	ND	ND	**ND**	ND	ND
			♀	**ND**	ND	ND	**ND**	ND	ND	**4**	8.50	3.40	**14**	5.91	3.43	**5**	8.60	4.27	**ND**	ND	ND	**ND**	ND	ND
	Pushing force endurance of the arms and core stability	Push-ups, No.	All	**49**	12.4	3.0	**52**	14.0	2.5	**84**	15.9	3.9	**82**	16.1	3.5	**22**	15.8	3.6	**ND**	**ND**	**ND**	**ND**	**ND**	**ND**
			♂	**27**	12.1	3.4	**31**	14.4	2.6	**47**	16.2	4.2	**58**	16.4	3.5	**17**	15.7	3.7	**ND**	ND	ND	**ND**	ND	ND
			♀	**22**	12.7	2.4	**21**	13.6	2.4	**37**	15.4	3.4	**24**	15.5	3.4	**5**	16.2	3.3	**ND**	ND	ND	**ND**	ND	ND
	APCT for validity check	APCT, m	All	**49**	2.54	1.80	**52**	3.96	3.68	**84**	5.89	4.16	**82**	7.15	4.55	**22**	5.77	4.04	**ND**	ND	ND	**ND**	ND	ND
			♂	**27**	2.83	1.94	**31**	3.63	2.77	**47**	6.08	3.92	**58**	7.61	4.47	**17**	6.12	4.28	**ND**	ND	ND	**ND**	ND	ND
			♀	**22**	2.17	1.58	**21**	4.45	4.75	**37**	5.64	4.50	**24**	6.05	4.65	**5**	4.59	3.21	**ND**	ND	ND	**ND**	ND	ND
Lower body strength	muscle strength of the legs	SLJ, m	All	**50**	122.1	18.0	**77**	148.4	20.3	**105**	157.9	23.1	**83**	174.9	26.1	**157**	202.4	27.7	**105**	188.2	40.8	**40**	194.6	44.0
			♂	**27**	131.0	15.5	**50**	153.9	16.0	**62**	163.3	18.7	**58**	181.1	24.5	**127**	210.4	23.0	**61**	211.5	32.1	**27**	215.4	32.6
			♀	**23**	111.8	15.2	**27**	138.2	23.6	**43**	150.2	26.7	**25**	160.6	24.5	**30**	168.6	19.0	**44**	155.8	27.0	**13**	151.5	31.8
	APCT for validity check	APCT, m	All	**50**	2.50	1.80	**77**	4.69	3.88	**105**	5.47	4.07	**83**	7.20	4.55	**157**	7.98	4.61	**105**	7.30	5.26	**40**	7.52	4.34
			♂	**27**	2.83	1.94	**50**	4.40	3.18	**62**	5.55	3.81	**58**	7.61	4.47	**127**	8.62	4.54	**61**	9.39	5.36	**27**	9.53	3.60
			♀	**23**	2.11	1.58	**27**	5.24	4.95	**43**	5.37	4.46	**25**	6.27	4.68	**30**	5.25	3.90	**44**	4.41	3.48	**13**	3.36	2.29

N = Study population, Data are Mean and SD.; No. = number, SD = standard deviation, kg = kilogram, cm = centimeter, kg/kg-bw = Kilogram per kilogram of body weight, m = Meter, MHF – R = maximum Hand force – right Hand, MHF – L = maximum Hand force – left Hand, PU – OG = Pull-ups - over grip, PU – UG = Pull-ups - under grip, APCT = Austrian Pole Climbing Test, ♂ = Boys, ♀ = Girls, ND = no data.

Table S13. Age-specific data from the validity check using Pearson's 's correlation coefficient

Age group	Upper body strength						Lower body strength		
	Maximum hand force, pulling force endurance of the arms			Pushing force endurance of the arms and core stability			muscle strength of the legs		
	**Group**	**Variable**	**APCT, rs**	**Group**	**Variable**	**APCT, rs**	**Group**	**Variable**	**APCT, rs**
**≤ 7**	**ND**	**MHF - R, kg/kg-bw**	ND	**All (N=49)**	**Push-ups, No.**	.209	**All (N=50)**	**SLJ, cm**	.475**
		**MHF - L, kg/kg-bw**							
		**PU - OG, No.**							
		**PU - UG, No.**							
**8 to 9**	**ND**	**MHF - R, kg/kg-bw**	ND	**All (N=52)**	**Push-ups, No.**	.448**	**All (N=77)**	**SLJ, cm**	.413**
		**MHF - L, kg/kg-bw**							
		**PU - OG, No.**							
		**PU - UG, No.**							
**10 to 11**	**All (N=17)**	**MHF - R, kg/kg-bw**	.598*	**All (N=84)**	**Push-ups, No.**	.383**	**All (N=105)**	**SLJ, cm**	.465**
		**MHF - L, kg/kg-bw**	.480						
		**PU - OG, No.**	.755**						
		**PU - UG, No.**	.580**						
**12 to 13**	**All (N=55)**	**MHF - R, kg/kg-bw**	.562**	**All (N=82)**	**Push-ups, No.**	.292**	**All (N=83)**	**SLJ, cm**	.495**
		**MHF - L, kg/kg-bw**	.581**						
		**PU - OG, No.**	.725**						
		**PU - UG, No.**	.684**						
**14 to 15**	**All (N=17)**	**MHF - R, kg/kg-bw**	.608**	**All (N=22)**	**Push-ups, No.**	.509*	**All (N=157)**	**SLJ, cm**	.547**
		**MHF - L, kg/kg-bw**	.432						
		**PU - OG, No.**	.799**						
		**PU - UG, No.**	.849**						
**16 to 17**	**ND**	**MHF - R, kg/kg-bw**	ND	**ND**	**Push-ups, No.**	ND	**All (N=105)**	**SLJ, cm**	.621**
		**MHF - L, kg/kg-bw**							
		**PU - OG, No.**							
		**PU - UG, No.**							
**18 to 19**	**ND**	**MHF - R, kg/kg-bw**	ND	**ND**	**Push-ups, No.**	ND	**All (N=40)**	**SLJ, cm**	.698**
		**MHF - L, kg/kg-bw**							
		**PU - OG, No.**							
		**PU - UG, No.**							

Data are Pearson's 's correlation coefficients: The strength of the correlations was classified according to Cohen, whereby a weak correlation was classified as ≥ 0.1, a moderate correlation as ≥ 0.3 and a strong correlation as ≥ 0.5; * = Correlation is significant at the 0.05 level (2-tailed); ** = Correlation is significant at the 0.01 level (2-tailed); kg = kilogram, cm = centimeter, kg/kg-bw = Kilogram per kilogram of body weight, m = Meter, MHF – R = maximum Hand force – right Hand, MHF – L = maximum Hand force – left Hand, PU – OG = Pull-ups - over grip, PU – UG = Pull-ups - under grip, APCT = Austrian Pole Climbing Test, ♂ = Boys, ♀ = Girls, ND = no data.

Table S14. Checking competitiveness using Pearson's 's correlation coefficient

Age group	Variable	Variable	APCT, m	SPC, sec
**All**	**All (N=82)**	**APCT, m**	--	
		**SPC, sec**	-.594**	--
	**♂ (N=59)**	**APCT, rs**	--	
		**SPC, sec**	-.610**	--
	**♀ (N=23)**	**APCT, rs**	--	
		**SPC, sec**	-.521*	--

Data are Pearson's 's correlation coefficients: The strength of the correlations was classified according to Cohen, whereby a weak correlation was classified as ≥ 0.1, a moderate correlation as ≥ 0.3 and a strong correlation as ≥ 0.5; * = Correlation is significant at the 0.05 level (2-tailed); ** = Correlation is significant at the 0.01 level (2-tailed); m = Meter, sec = seconds, APCT = Austrian Pole Climbing Test, SPC = Speed Pole Climbing, ♂ = Boys, ♀ = Girls.

Figure S1. Achievements versus No- Achievements at the Austrian Pole Climbing Test by the total study population and for subgroups boys (♂) and girls (♀)

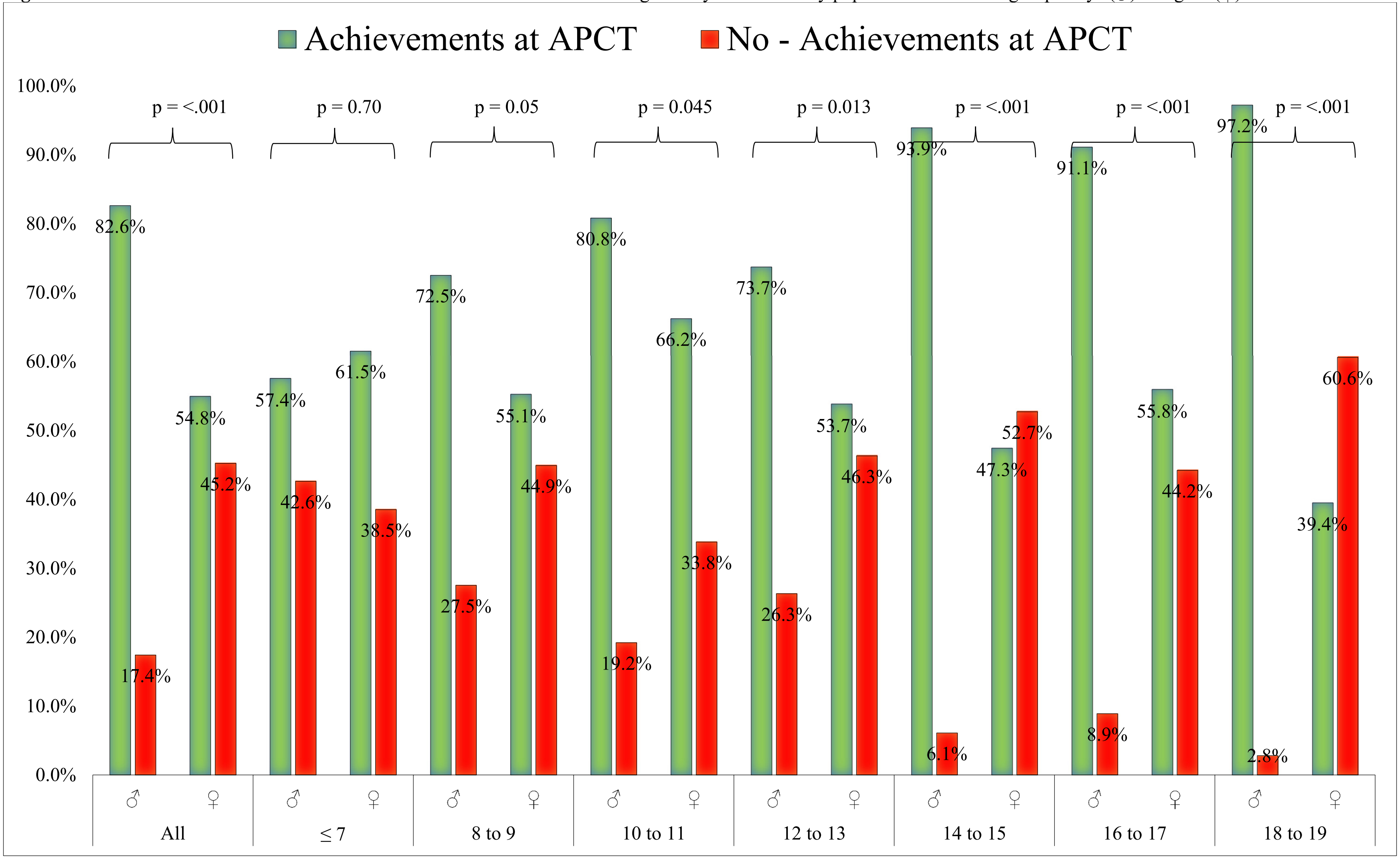

Figure S2. Gender and age-specific mean values of raw values of the Austrian Pole Climbing Test

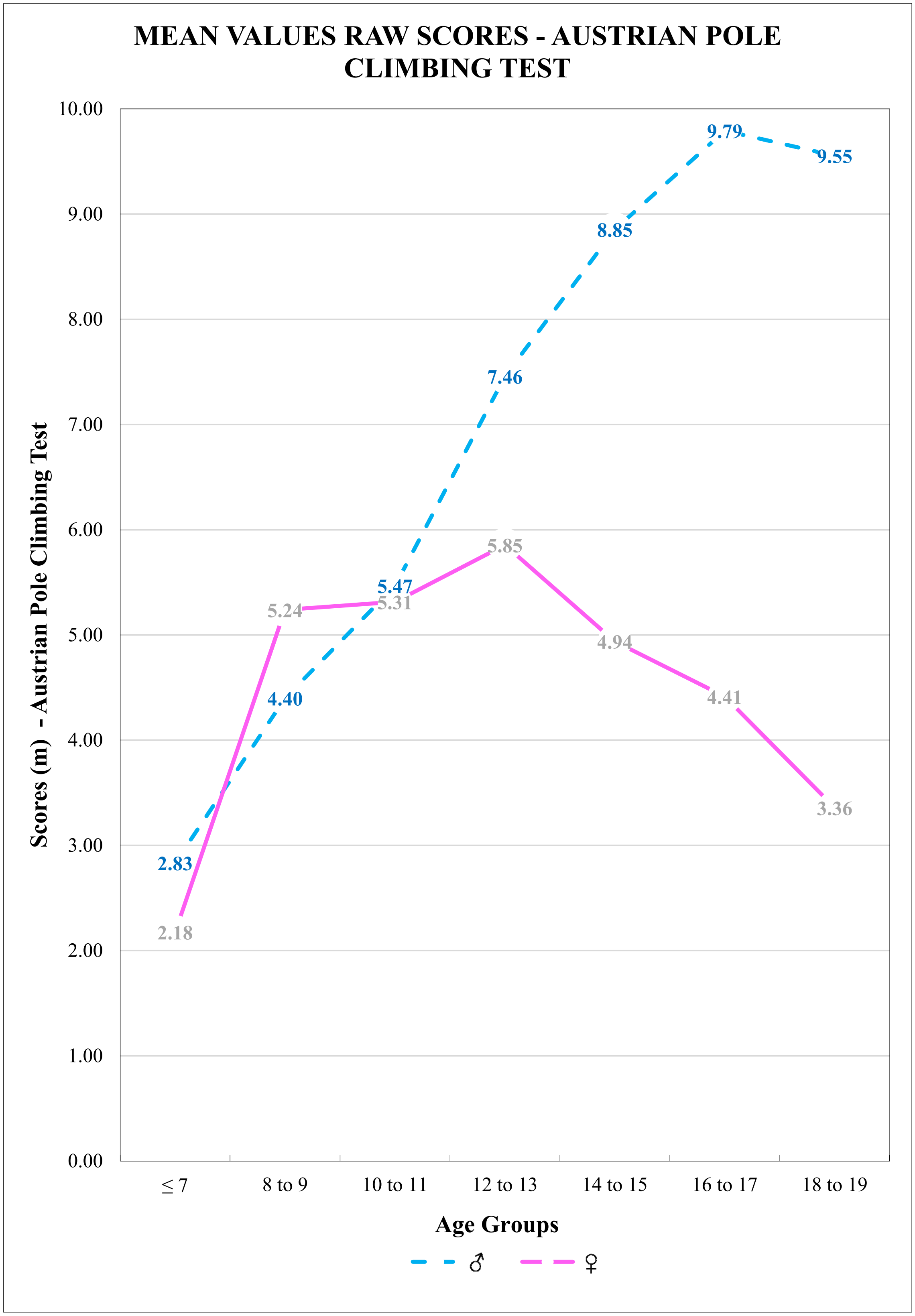

Figure S3. Graphical checking of normal distribution using histograms for classification of Austrian Pole Climbing Test and an established Speed Pole Climbing Test.

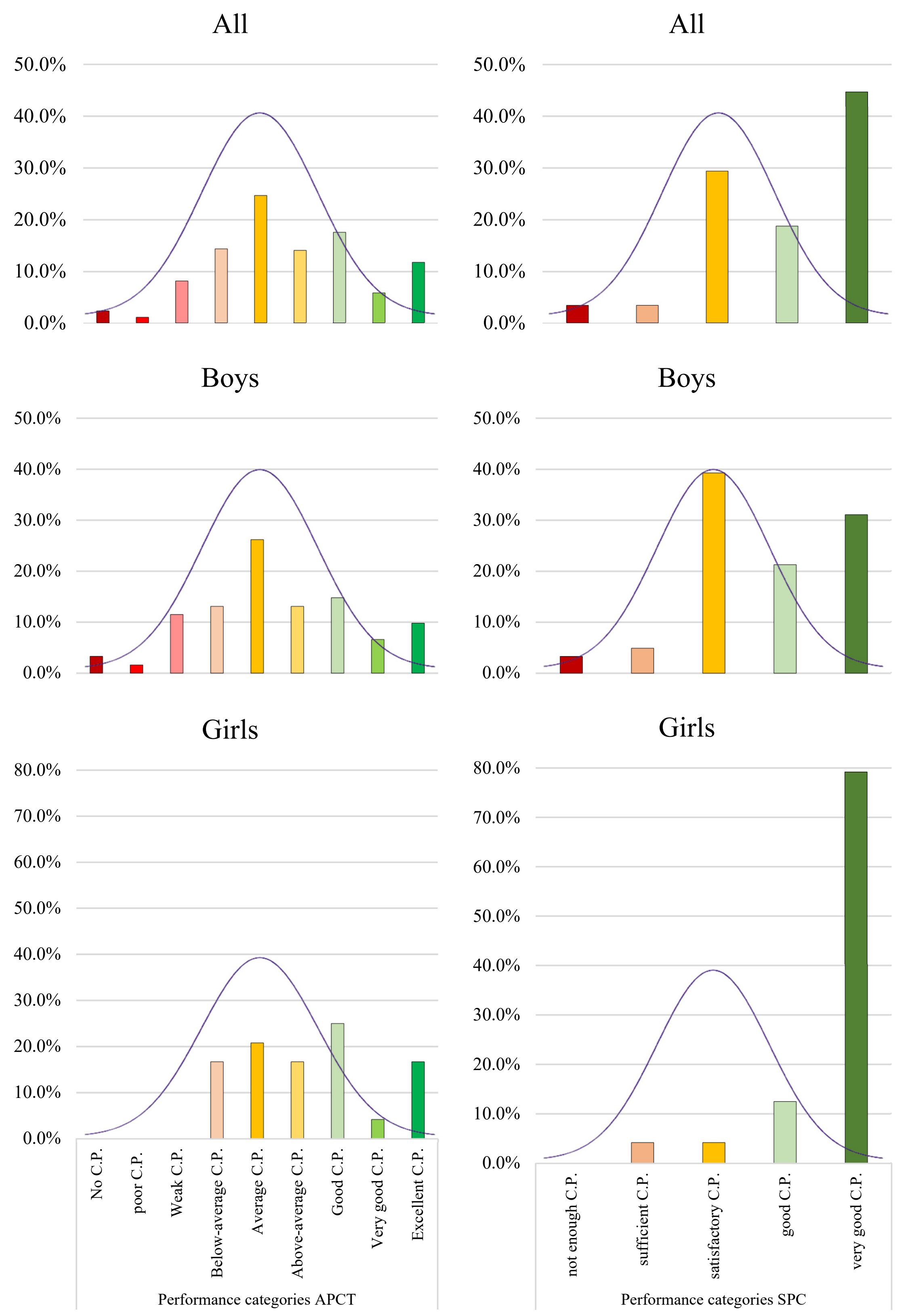

C.P. = Climbing Performance, APCT = Austrian Pole Climbing Test, SPC = Speed Pole Climbing.

REFERENCES

1 Mayer M, Gleiss A, Häusler G, et al. Weight and body mass index (BMI): current data for Austrian boys and girls aged 4 to under 19 years. *Ann Hum Biol* 2015;42(1):45–55.

2 Cole TJ, Bellizzi MC, Flegal KM, et al. Establishing a standard definition for child overweight and obesity worldwide: international survey. *BMJ* 2000;320(7244):1240–43.
